# Nitro‐fatty acids‐mediated nitroalkylation modulates fine‐tuning catalase antioxidant function during salinity stress in plants

**DOI:** 10.1002/pro.70076

**Published:** 2025-02-25

**Authors:** Mounira Chaki, Lorena Aranda‐Caño, Juan C. Begara‐Morales, Beatriz Sánchez‐Calvo, Francisco Javier López‐Jaramillo, María N. Padilla, Raquel Valderrama, José Rafael Pedrajas, Juan B. Barroso

**Affiliations:** ^1^ Group of Biochemistry and Cell Signaling in Nitric Oxide, Department of Experimental Biology, Faculty of Experimental Sciences University Institute of Research in Olive Groves and Olive Oils, Campus Las Lagunillas, University of Jaén Jaén Spain; ^2^ Department of Biochemistry and Biomedical Research Center (CEINBIO), School of Medicine University of the Republic Montevideo Uruguay; ^3^ Department of Basic Nutrition, School of Nutrition University of the Republic Montevideo Uruguay; ^4^ Institute of Biotechnology University of Granada Granada Spain

**Keywords:** abiotic stress, *Arabidopsis thaliana*, catalase, nitric oxide, nitroalkylation, nitro‐fatty acids, nitro‐linolenic acid, post‐translational modification, salinity stress, signaling

## Abstract

Nitro‐fatty acids (NO_2_‐FAs) are novel molecules resulting from the interaction of unsaturated fatty acids and nitric oxide (NO) or NO‐related molecules. In plants, it has recently been described that NO_2_‐FAs trigger a powerful antioxidant and defense response against stressful situations, the induction of the heat‐shock response (HSR), and they exert their signaling function mainly through a reversible post‐translational modification called nitroalkylation. Catalase (CAT) is a key antioxidant enzyme for the control of the hydrogen peroxide (H_2_O_2_) levels generated by environmental oxidative stress. The data presented in this study provide novel information on the role of NO_2_‐FAs in modulating the antioxidant activity of catalase 2 (CAT2) during salinity stress in *Arabidopsis thaliana*. Initially, in vitro treatment with nitro‐linolenic acid (NO_2_‐Ln) down‐regulated Arabidopsis CAT2 activity, as a consequence of the nitroalkylation of His 156 and His 248, evolutionarily conserved residues with key functional implications for the quaternary structure and hence CAT2 activity. Any effect of NO_2_‐Ln on the heme group or *S*‐nitrosylation of CAT2 was excluded. To further our knowledge of the regulatory mechanism of this antioxidant enzyme by nitroalkylation, the functional modulation of CAT by NO_2_‐FAs was analyzed in 5‐day‐old Arabidopsis cell suspension cultures subjected to salinity stress. In this situation, the oxidative stress generated caused the nitroalkylation of these residues to disappear through the cleavage of NO_2_‐Ln binding to CAT2, thus restoring CAT2 catalytic activity. Thus, during salinity stress, CAT2 enzymatic activity increased without changes in protein levels. These results highlight the amino acid targets that are susceptible to nitroalkylation and the modulatory effect of this post‐translational modification on CAT2 enzymatic activity in vitro and in vivo. These findings underline the regulatory role of nitroalkylation in CAT2 functionality, which is strongly influenced by the redox state thus becoming a new key control mechanism of this antioxidant enzyme in abiotic stress cell response processes.

## 1 INTRODUCTION

Salinity is an environmental stress factor that harmfully affects plant crop yields and productivity. To persist in high soil salinity stress, plants develop many strategies, including stomatal closure, control of the Na^+^ efflux across the plasma membrane, and ROS scavenging (Siddiqui et al. 2011). In plant species, sodium chloride (NaCl) reduces leaf fresh weight and produces an imbalance between reactive oxygen species (ROS) production and scavenging, which in turn leads to oxidative stress (Begara‐Morales et al. 2014; Tanou et al. 2012; Valderrama et al. 2006). To protect themselves, plant cells have developed strategies to maintain redox homeostasis. These strategies include scavenging ROS or activating the non‐enzymatic and enzymatic antioxidants network. In this context, catalase (CAT) is one of the most important antioxidant enzymes in plants and catalyzes the dismutation of hydrogen peroxide (H_2_O_2_) into water and oxygen molecules to regulate ROS levels (Mhamdi et al. 2010; Willekens et al. 1997). In leaf tissue, CAT is located in peroxisomes, where it functions to remove the H_2_O_2_ produced by glycolate oxidase in the photorespiratory cycle C2. The three catalases identified in the Arabidopsis genome exhibit a high degree of similarity, with identity levels ranging from 75 to 84%. The available evidence suggests that Arabidopsis CAT1, CAT2, and CAT3 are below Class III, Class I, and Class II catalases, respectively. The CAT1 gene expression level was found to be minimal, with its activity being undetectable. Arabidopsis CAT2, on the other hand, possesses approximately 80% of the total CAT activity (Chen et al. 2020).

Nitrated lipids, or nitro‐fatty acids (NO_2_‐FAs), are endogenously generated from the non‐enzymatic oxidation of unsaturated fatty acids by nitric oxide (NO)‐related species (Freeman et al. 2008). NO_2_‐FAs are present at low concentrations in yeasts, animals, humans, and plant systems (Aranda‐Caño et al. 2022a; Aranda‐Caño et al. 2022b; Baker et al. 2004; Balazy and Poff 2004; Begara‐Morales et al. 2021; Mata‐Pérez et al. 2016b; Mata‐Pérez et al. 2017; Tsikas et al. 2011). In animal cells, NO_2_‐FAs are molecules that exhibit pleiotropic effects, which translate into cytoprotective actions in a wide range of diseases, such as atherosclerosis, ischemia–reperfusion, kidney damage, diabetes, dermatitis, obesity, and cancer (Brat et al. 2023; Chartoumpekis et al. 2024; Rom et al. 2020; Sánchez‐Calvo et al. 2016; Wang et al. 2016; Wang et al. 2021; Zhao et al. 2024; Zhou et al. 2021). In yeast cells, nitro‐oleic acid (NO_2_‐OA) modulates Tsa1 protein activity during heat stress (Aranda‐Caño et al. 2022b). The presence of nitro‐conjugated linoleic acid (NO_2_‐cLA) has been reported in plant organisms for the first time in olive and extra virgin olive oil. The anti‐inflammatory properties of olive oil are associated with the presence of this compound (Fazzari et al. 2014). Subsequently, NO_2_‐OA, nitro‐linoleic acid (NO_2_‐LA), and nitro‐linolenic acid (NO_2_‐Ln) have been detected in Arabidopsis plants during development (Aranda‐Caño et al. 2022a). Furthermore, NO_2_‐Ln has been identified in cell suspension cultures (Mata‐Pérez et al. 2017), and in response to adverse environmental factors in Arabidopsis (Mata‐Pérez et al. 2016c), in leaves and in roots of rice plants, peroxisomes, and mitochondria of pea plants (Mata‐Pérez et al. 2017). Subsequent studies have identified that the presence of NO_2_‐OA was detected in *Brassica napus* (Vollár et al. 2020).

Furthermore, the ability of NO_2_‐FAs to act as NO donors has been described in both plants and animals (Gorczynski et al. 2006; Mata‐Pérez et al. 2016a; Vollár et al. 2020). NO released from NO_2_‐FAs is considered to be a key modulator of several cellular processes. In human leukemia cells and rats, NO_2_‐LA *S*‐nitrosylates the CD40 protein, thereby inducing an anti‐inflammatory response (Faine et al. 2010). Likewise, it has been shown that the NO released from NO_2_‐Ln has the capacity to synthesize *S*‐nitrosoglutathione (GSNO) in the presence of glutathione (GSH) and to modulate its levels both in vitro and in vivo, as well as indirectly modulating *S*‐nitrosothiol levels (Mata‐Pérez et al. 2020). NO_2_‐Ln‐dependent *S*‐nitrosylation of the transcription factor bZIP67, which is a master regulator in seed maturation programmes, has recently been described to modulate the lipid accumulation profile during embryonic development (Sánchez‐Vicente et al. 2024). Therefore, the ability of NO_2_‐FAs to act as signaling molecules through released NO cannot be excluded.

NO_2_‐FAs have powerful electrophilic reactivity due to the presence of electrons by drawing nitro group substituents in β‐carbon. These molecules have the capacity to adduct mainly with the cysteine (Cys), histidine (His), and lysine (Lys) amino acids of proteins through a reversible post‐translational modification (PTM) named nitroalkylation, which affects protein structure, cellular localization, and protein function (Aranda‐Caño et al. 2019; Aranda‐Caño et al. 2022b; Rudolph et al. 2009). In animal cells, NO_2_‐OA and NO_2_‐LA have the capacity to modulate the protein function of Nrf2/Keap1, NFκβ, and PPAR‐γ by nitroalkylation modification and to, subsequently, promote anti‐inflammatory reactions (Schopfer et al. 2011; Schopfer and Khoo 2019). Interestingly, the increased levels of H_2_O_2_ and peroxynitrite (ONOO^−^) generated under nitro‐oxidative stress conditions in abiotic stress factors can release NO_2_‐FAs from adducted proteins. In this way, the protein regains its function, and free NO_2_‐FA exerts its signaling effect (Aranda‐Caño et al. 2022b).

Due to the difficulty of identifying nitroalkylated target proteins and their modified sites by mass spectrometry, this new PTM has been little studied, although some candidate proteins have been identified (Aranda‐Caño et al. 2019; Aranda‐Caño et al. 2022b; Fang et al. 2021). This PTM needs to be further characterized to define its relevance in plant physiology. CAT is a heme antioxidant enzyme that could act as a putative target of nitroalkylation, similar to what has been described for other antioxidant proteins like cytosolic ascorbate peroxidase 2 (APX2), which was found to be nitroalkylated in NO_2_‐Ln‐treated Arabidopsis cell cultures (Aranda‐Caño et al. 2019), or Tsa1 nitroalkylated by NO_2_‐OA in *Saccharomyces cerevisiae* (Aranda‐Caño et al. 2022b). To increase our knowledge of the regulatory mechanism of this antioxidant enzyme by nitroalkylation, the functional modulation of CAT by NO_2_‐FAs was analyzed in 5‐day‐old Arabidopsis cell suspension cultures treated with 150 mM NaCl. Nitroalkylated residues, involved in the modulation of CAT activity, were identified and their functional involvement was reported. Nitroalkylation may act as another key PTM to adjust the structure and function of this heme protein, similar to what has been described for other PTMs like phosphorylation, acetylation, nitration, and *S*‐nitrosylation, among others (Lin 2018).

## 2 RESULTS

### 2.1 Down‐regulation of catalase activity by NO_2_ ‐Ln

Recently, it has been reported that post‐translational modification, specifically nitroalkylation by NO_2_‐FAs, has the capacity to modulate the catalytic activity of plant proteins (Aranda‐Caño et al. 2019; Aranda‐Caño et al. 2022b). In order to evaluate the potential effect of NO_2_‐Ln on catalase activity, an in vitro assay was carried out using different catalase sources, including Arabidopsis leaf peroxisomes, Arabidopsis recombinant CAT2 protein, and CAT protein from bovine liver. These catalase sources showed similar behavior in the presence of NO_2_‐FAs.

**FIGURE 1:**
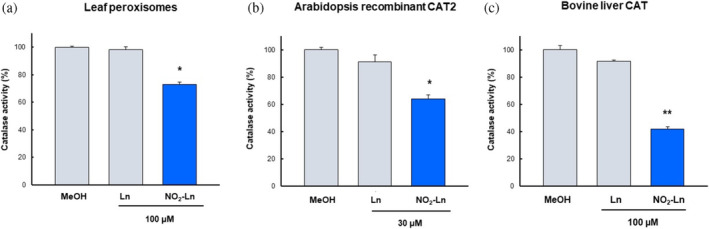
NO_2_‐Ln treatment inhibits catalase activity in Arabidopsis leaf peroxisome (a), recombinant CAT2 (b), and CAT from bovine liver (c). The samples were treated with control vehicle (MeOH), Ln (non‐nitrated form of NO_2_‐Ln), and NO_2_‐Ln. The specific catalase activity of leaf peroxisomes was determined to be 376.6 ± 1.9 mU H_2_O_2_ mg⁻^1^ protein, 369.8 ± 5.1 mU H_2_O_2_ mg⁻^1^ protein, and 273.8 ± 4.9 mU H_2_O_2_ mg⁻^1^ protein, respectively, corresponding to MeOH, Ln, and NO_2_‐Ln. Recombinant CAT2 activity from Arabidopsis was 15235.3 ± 328.3 mU H_2_O_2_ mg⁻^1^ protein 12823.7 ± 363.3 mU H_2_O_2_ mg⁻^1^ protein, and 9723.5 ± 265.6 mU H_2_O_2_ mg⁻^1^ protein, respectively, corresponding to MeOH, Ln, and NO_2_‐Ln. Finally, the specific catalase activity of bovine liver was 6.18 ± 0.1 mU H_2_O_2_ mg⁻^1^ protein, 5.67 ± 0.05 mU H_2_O_2_ mg⁻^1^ protein, and 2.6 ± 0.11 mU H_2_O_2_ mg⁻^1^ protein, respectively, corresponding to MeOH, Ln, and NO_2_‐Ln. The results are means ± SEM of at least three independent experiments. Statistical significant differences *p* < 0.05 (*) and *p* < 0.001 (**) from the control. NO_2_‐Ln: nitro‐linolenic acid; Ln: linolenic acid.

Thus, as shown in Figure 1, catalase activity was significantly down‐regulated in leaf peroxisomes, Arabidopsis recombinant protein, and bovine liver, with respective reductions of 26%, 36%, and 60%.

### 2.2 Evaluation of the heme content of NO_2_ ‐Ln‐treated catalase by the pyridine hemochrome method

All catalase isoforms contain a heme group, which is essential for their enzymatic activity (Padovani et al. 2016). To determine whether NO_2_‐Ln‐mediated catalase inhibition involves the heme group, the stability of the heme group of NO_2_‐Ln‐treated catalase was assessed by monitoring the spectrum of pyridine haemochromogen by spectrophotometry in the scanning mode from 350 to 700 nm (Berry and Trumpower 1987; Trostchansky et al. 2011).

As shown in Figure S1, Supporting Information, the NO_2_‐Ln treatment with the Arabidopsis recombinant CAT2 protein did not change the spectrum of the pyridine hemochromogen compared to the sample treated with the NO_2_‐FA vehicle (methanol) (Figure S1a). This established that there was neither a direct reaction of NO_2_‐FA with heme nor any displacement of this molecule from CAT2. Similar results were obtained using bovine liver catalase (Figure S1b). As a control, the reaction of commercial heme (Sigma) with NO_2_‐Ln did not alter the spectrum of the heme group (Figure S1c).

### 2.3 In vitro *S*‐nitrosylation of the recombinant CAT2 protein by NO_2_ ‐Ln

**FIGURE 2:**
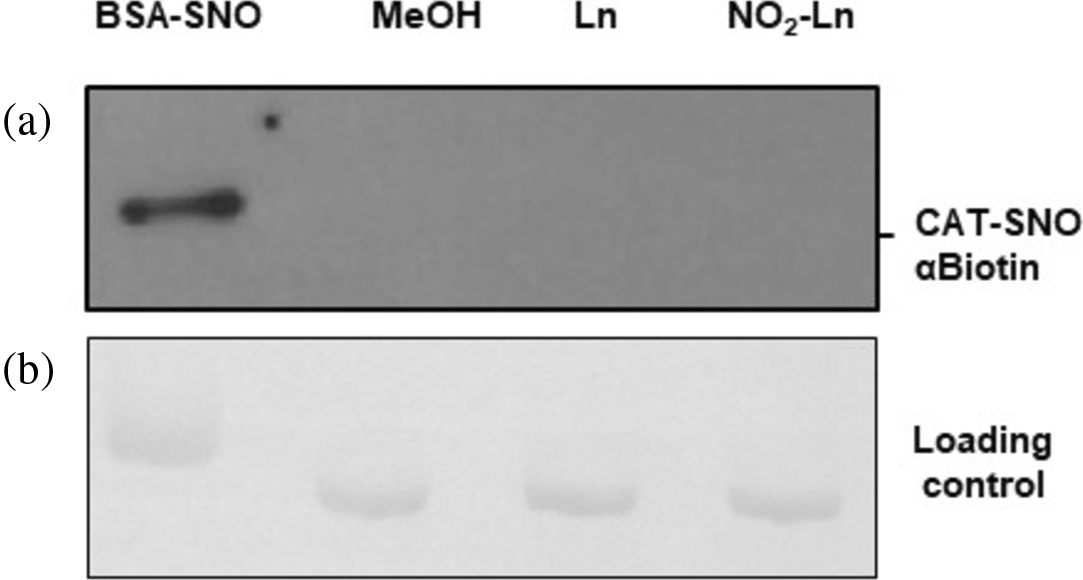
(a) Arabidopsis recombinant CAT 2 protein is not a target of NO derived from NO_2_‐Ln. As a control of the biotin switch method, bovine serum albumin (BSA) was *S*‐nitrosylated with 500 μM NO_2_‐Ln (Line 1). Control treatments were done with the vehicle (MeOH) (lane 2) and 500 μM Ln (lane 3). Five microgram of each proteins were separated using a 10% non‐reducing SDS‐PAGE and blotted onto a PVDF membrane. Biotinylated proteins were identified by anti‐biotin antibody. NO: nitric oxide; NO_2_‐Ln: nitro‐linolenic acid; Ln: linolenic acid; MeOH: methanol. (b) Ponceau staining ensures equal protein loading.

NO_2_‐Ln has the capacity to act as a NO donor under physiological conditions (Mata‐Pérez et al. 2016a; Mata‐Pérez et al. 2020) and this NO can exert its function through protein *S*‐nitrosylation. In order to study the effect of NO_2_‐Ln on antioxidant protein function, Arabidopsis recombinant CAT2 protein was subjected to a biotin switch assay. As shown in Figure 2a, the treatment of recombinant CAT2 protein with 500 μM NO_2_‐Ln (lane 4) did not result in a *S*‐nitrosylated band. As negative controls, recombinant CAT2 protein was treated with the vehicle (MeOH) (lane 2), and also with 500 μM linolenic acid (Ln) (lane 3), but no signal was observed. As a positive control, bovine serum albumin protein (Sigma) was incubated with 500 μM NO_2_‐Ln, and a reactive band was detected. Regulation of CAT2 protein by the *S*‐nitrosylation process of NO_2_‐Ln was therefore excluded.

### 2.4 Detection of nitroalkylated peptides and the relative quantification of the nitroalkylated residues in recombinant CAT2 protein

**FIGURE 3:**
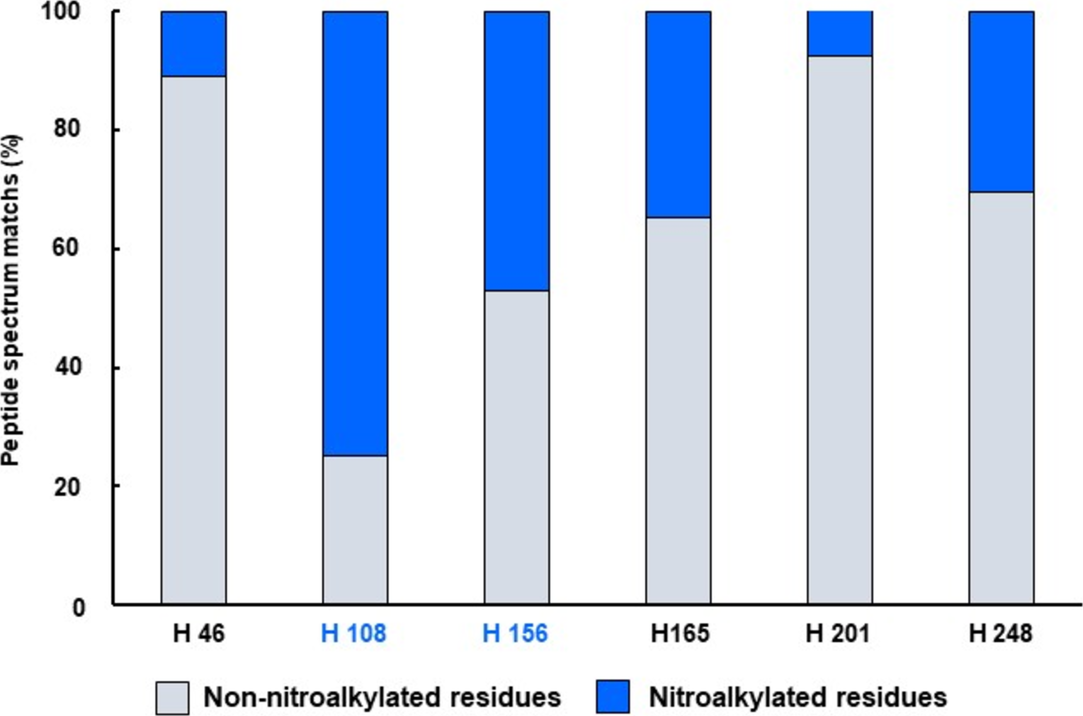
Detection of nitroalkylated residues in Arabidopsis recombinant CAT2 protein treated with 350 μM NO_2_‐Ln by nano‐LC‐MS/MS. Relative percentage of the peptide spectrum matches (PSMs) of the nitroalkylated and non‐nitroalkylated residues.

Another mechanism of action of NO_2_‐FAs is via nitroalkylation modification (Aranda‐Caño et al. 2019; Aranda‐Caño et al. 2022b; Trostchansky et al. 2011). Following the exclusion of the functional modulation of the CAT2 protein by *S*‐nitrosylation, an investigation was conducted into the nitroalkylation of the CAT2 protein by NO_2_‐Ln. Recombinant CAT2 protein was treated with NO_2_‐Ln, digested with trypsin, and analyzed by nano‐LC‐MS/MS. Six nitroalkylated peptides were detected, and the modified residues were identified (Table 1). The relative quantification of the nitroalkylated residues was performed by taking into account the number of peptide spectrum matches (PSMs) detected after a directed search of the nitroalkylated and non‐nitroalkylated peptides in the mass spectrometer. As shown in Figure 3, the residues that were highly nitroalkylated were His 108 and His 156, followed by His 248 and His 165. The residues that were slightly nitroalkylated were His 46 and His 201.

**TABLE 1:** Nano‐LC‐MS/MS detection of the nitroalkylated peptides and the identification of the modified residues in the Arabidopsis recombinant CAT2 protein treated with 350 μM NO_2_‐Ln.

Nitroalkylated peptides	Length (amino acids)	Nitroalkylated residues
GPILLEDY**H**LVEK	13	His 46
FSTVI**H**ER	08	His 108
FPDMV**H**ALKPNPK	13	His 156
S**H**IQENWR	08	His 165
**H**MDGSGVNTYMLINK	15	His 201
VGGTN**H**SHATQDLYDSIAAGNYPEWK	26	His 248

*Note*: The position of the nitroalkylated residues is shown in bold.

Abbreviation: His, histidine.

### 2.5 Effect of H_2_O_2_ on the nitroalkylation of recombinant CAT2 protein

Under both oxidative and nitrosative stress conditions, oxidation of the Michael adduct can occur, resulting in the release of NO_2_‐FA bound to the protein by nitroalkylation (Aranda‐Caño et al. 2022b; Baker et al. 2009; Mata‐Pérez et al. 2016c; Padilla et al. 2017; Schopfer and Khoo 2019). An experimental design similar to the work of Padilla et al. (2017) is used to demonstrate the effect of H_2_O_2_ (representative molecule for oxidative stress) on the NO_2_‐Ln‐mediated nitroalkylation of the recombinant CAT2 protein of Arabidopsis. Specifically, the occurrence of nitroalkylation in recombinant CAT2 protein by NO_2_‐Ln was examined by targeted mass spectrometry techniques before and after treatment with 1.5 mM H_2_O_2_ for 200 min. For this purpose, targeted mass analysis was performed for the peptide FSTVIHER, which contains histidine 108. This residue is the most susceptible to nitroalkylation by NO_2_‐Ln among the recombinant CAT2 protein. A targeted search for nitroalkylated and non‐nitroalkylated FSTVIHER peptides enabled the relative quantification of nitroalkylation before and after treatment with H_2_O_2_. Analysis of the data demonstrated that treatment of the recombinant CAT2 protein with NO_2_‐Ln resulted in a significant presence of nitroalkylated His 108 in the FSTVIHER peptide. However, treatment with H_2_O_2_ for 200 min showed the complete disappearance of the nitroalkylated His 108, with the peptide being detected without the mass increase generated by NO_2_‐Ln binding (Figure S2).

### 2.6 H_2_O_2_ levels and CAT2 protein activity under salinity stress

Salinity stress is a type of abiotic stress that increases ROS and NO levels, leading to oxidative and nitrosative stress in many plant species (Munns and Tester 2008; Valderrama et al. 2007). In addition, a relationship between salinity tolerance and fatty acid unsaturation levels has been described after it was shown that overexpression of ω‐3 desaturases (which increase Ln levels) increases salinity tolerance (Zhang et al. 2005). Therefore, this type of abiotic stress was chosen to analyze the relevance of CAT nitroalkylation in vivo.

**FIGURE 4:**
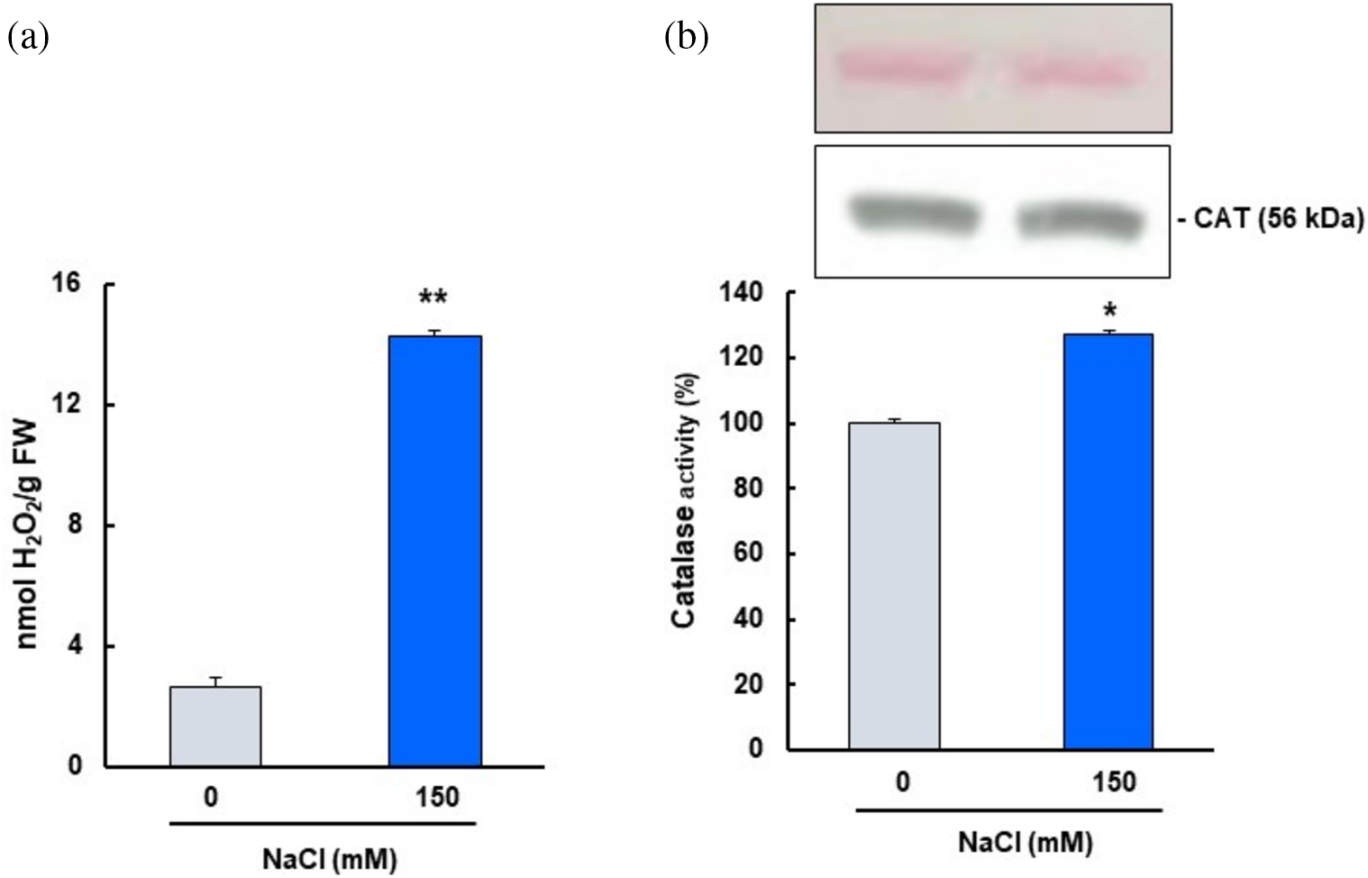
Salinity stress increased hydrogen peroxide levels and CAT activity in 5‐day‐old Arabidopsis cell cultures. (a) H_2_O_2_ content in Arabidopsis cell cultures control and treated with 150 mM NaCl. (b) CAT activity and immunoblot analysis using an antibody against CAT (dilution 1:1000). The Ponceau was included as a loading control. Results are means ± SEM of at least three replicates. Statistical significant differences *p* < 0.05 (*) and *p* < 0.001 (**) from control values. H_2_O_2_: hydrogen peroxide; NaCl: sodium chloride; FW: fresh weight.

Accordingly, 5‐day‐old Arabidopsis cell cultures were treated with 150 mM NaCl for 5 min, and the occurrence of oxidative stress was detected by measuring H_2_O_2_ levels. As shown in Figure 4a, salinity stress resulted in a 5.3‐fold increase in H_2_O_2_ levels.

In the same situation, CAT activity was also assessed. NaCl treatment resulted in a 27% increase in CAT activity (Figure 4b), which was not due to a rise in CAT protein content. Immunoblot analysis using an anti‐catalase antibody to determine CAT protein content showed no significant changes with saline treatment, and Ponceau staining of the membrane indicated equal loading (Figure 4b).

### 2.7 Modulation of NO_2_ ‐Ln levels during salinity stress

**FIGURE 5:**
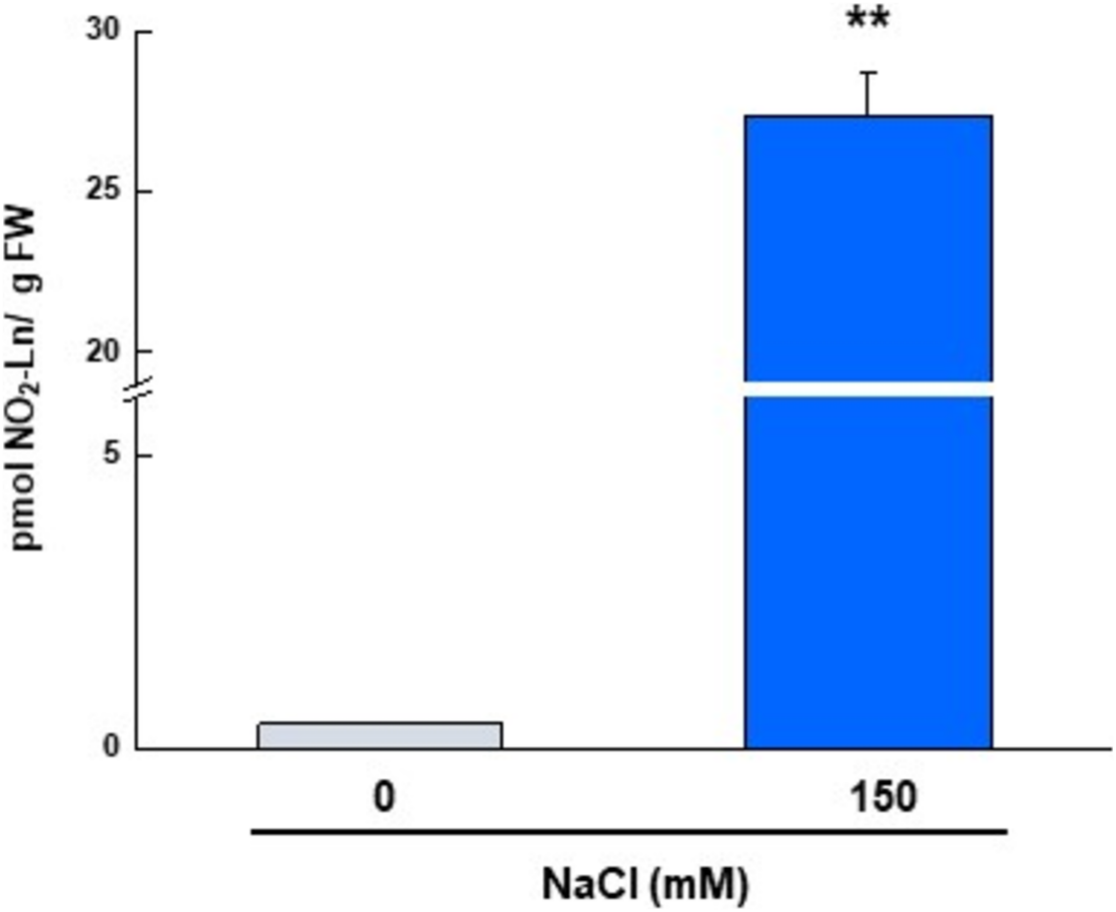
Salinity stress increased the endogenous levels of NO_2_‐Ln in 5‐day‐old Arabidopsis cell cultures. NO_2_‐Ln levels were detected by LC‐MS/MS in cell cultures control and treated with 150 mM NaCl. The results are the mean ± SEM of at least three replicates. Statistical significant differences *p* < 0.001 (**) from control values. FW: fresh weight.

The endogenous presence of NO_2_‐Ln was determined in the 5‐day‐old Arabidopsis cell cultures, control and treated with 150 mM NaCl for 5 min. The lipid extraction samples were measured by LC‐MS/MS by monitoring the 322/46 and 322/275 transitions (*m*/*z*). NaCl treatment significantly increased NO_2_‐Ln levels (Figure 5). Similar data were reported in the 9‐day‐old Arabidopsis cell cultures treated with 150 mM NaCl for 5 and 30 min (Mata‐Pérez et al. 2016c).

### 2.8 Identification of the CAT2‐nitroalkylated peptides during salinity stress by nano‐LC‐MS/MS

To investigate whether protein nitroalkylation has a functional implication in vivo, the nitroalkylated targets were explored using both control and 150 mM NaCl‐treated Arabidopsis cell cultures. The nitroalkylated peptides identified in vitro in recombinant CAT2 protein were searched in the control and the salinity stress‐treated samples (Table S1). A bioinformatic search of the spectra of the precursor ions (MS1) from the nitroalkylated targets was done. Only two nitroalkylated targets were identified in the control sample, corresponding to His 156 and His 248. However, these targets disappeared under the salinity stress conditions (Table 2). These peptides appeared to be more susceptible to nitroalkylation in vivo and could therefore be interesting targets in response to salinity stress. These putative nitroalkylated targets were also identified in vitro (Table 1).

**TABLE 2:** Nano‐LC‐MS/MS identification of the catalase nitroalkylated residues in the Arabidopsis cell cultures control and the salinity‐treated samples.

Nitroalkylated peptides	Standard			Control			Salinity stress			
	Nitroalkylated targets in the recombinant CAT2 protein treated with NO_2_‐Ln			Nitroalkylated targets in the control Arabidopsis cell cultures			Nitroalkylated targets in the salinity‐treated Arabidopsis cell cultures			
	Nitroalkylated residues	RT	*m*/*z*	RT	*m*/*z*	*I*	RT	*m*/*z*	*I*
FPDMV**H**ALKPNPK	**His 156**	50.53	917.499	50.40	917.496	3.7 × 10^5^	ND	ND	ND
VGGTNHS**H**ATQDLYDSIAAGNYPEWK	**His 248**	58.84	1052.505	58.70	1052.505	1.59 × 10^4^	ND	ND	ND

*Note*: The RT and *m*/*z* parameters of the peptides identified in vitro in the recombinant CAT2 protein treated with NO_2_‐Ln, which were used as a standard. The RT, *m*/*z*, and *I* parameters of the peptides identified in vivo in the control and salinity stress samples. The position of the nitroalkylated residues is shown in bold.

Abbreviations: His, histidine; *I*, intensity; ND, not detected; RT, retention time.

### 2.9 Analysis of the residues involved in CAT2 nitroalkylation by docking studies

Nitroalkylation is a highly specific PTM that is both NO_2_‐FA and protein‐dependent. Not only the presence of regions of the protein with affinity for NO_2_‐FA, but also the chain length and the position of the nitroalkene group account for the selectivity of the PTM. Interestingly, different proteins show different selectivity for the distinct isomers of NO_2_‐FA. For example, of the four isomers of NO_2_‐LA, both carbon 10 (C10) and C12 isomers (i.e., nitro group at C10 or C12) have been reported to selectively bind to Cys 280 of PPARγ, with the C10 isomer being more reactive (Li et al. 2008; Schopfer et al. 2010).

To gain insight into CAT2 protein modulation by NO_2_‐Ln, docking of the six possible NO_2_‐Ln isomers was proposed as an approach to better understand the nitroalkylation of Arabidopsis CAT2. The blind docking results revealed that neither Lys residues nor Cys residues yielded feasible poses (i.e., those with no forbidden interaction and with the atoms forming the covalent bond between the protein and NO_2_‐Ln within the 4 Å radius).

Another residue that does not generate positive poses to be a nitroalkylation target in the docking result is His 108, despite being the residue most susceptible to nitroalkylation in the in vitro analysis of the treatment of recombinant CAT2 protein with NO_2_‐Ln. His 108 is located in a loop region close to the interface between the monomers (Figure S3a). This figure also shows the environment around His 108, which is characterized by a negative electrostatic potential (Figure S3c) and a polar environment (blue), despite being located between two hydrophobic patches (orange) (Figure S3b). Overall, both the hydrophilic residues and the negative electrostatic potential surrounding His 108 do not favor interaction with the NO_2_‐Ln alkyl chain. However, the fact that His 108 is located in a loop that confers a degree of flexibility and makes it susceptible to some degree of conformational change under in vitro assay conditions may provide an explanation for the high presence of this nitroalkylated residue in the mass spectrometric analyses performed after treatment of the recombinant CAT2 protein with NO_2_‐Ln (Figure 3).

**FIGURE 6:**
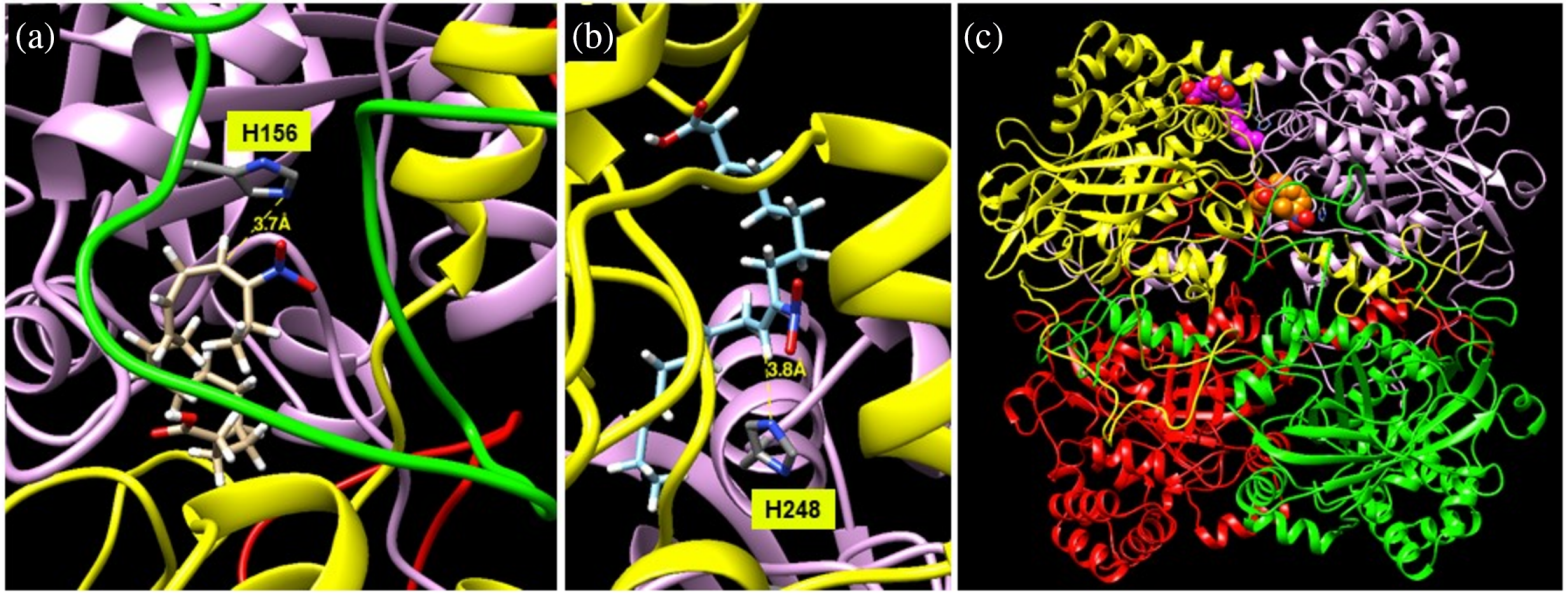
Pose of the docking of 16‐NO_2_‐Ln (a) and 9‐NO_2_‐Ln (b). Location of 16‐NO_2_‐Ln (orange) and 9‐NO_2_‐Ln (magenta) in the model of the quaternary structure of catalase from *A. thaliana* (c).

One of the positive poses was His 156 (Figure 6a), a residue detected as being nitroalkylated under physiological conditions, but not His 284. Local docking centered on the imidazole ring of His 284 indicated several poses that fulfilled the selection criteria (Figure 6b). Neither His 156 nor His 248 was located close to either the active site or the cofactor binding site to explain the inhibitory effect on catalysis. As enzyme regulation is a cornerstone of homeostasis, their evolutionary conservation in the *Viridiplanae clade* was estimated by determining the rho parameter (Mihalek et al. 2004). For both residues, rho is 1, as expected for absolutely conserved residues, confirming their important role in the enzyme. A closer analysis of the poses revealed that His 248 was located at the interface between two monomers and its nitroalkylation disrupted the interface, and probably the interaction between them (Figure 6c). The position of His 156 was even more relevant, and its nitroalkylation was more disruptive to the quaternary structure as it was located at the region protruding from the monomers and that involved in the interaction with the proximal upper and bottom monomers.

## 3 DISCUSSION

NO_2_‐FAs are interesting signaling molecules derived from the interaction of NO‐related molecules with unsaturated fatty acids (Begara‐Morales et al. 2021; Freeman et al. 2008; Khoo and Schopfer 2019). To date, their endogenous presence and some of their biological functions have been reported in plants (Aranda‐Caño et al. 2022a; Mata‐Pérez et al. 2016c; Vollár et al. 2020). Some biological functions act as a more stable NO storage potential in membrane lipid biomolecules (Aranda‐Caño et al. 2022a) given their ability to donate NO and to participate in the control of *S*‐nitrosothiol levels (Mata‐Pérez et al. 2020; Sánchez‐Vicente et al. 2024), and their potential as a genetic regulator of the stress response (Mata‐Pérez et al. 2016c). However, the effect of NO_2_‐FAs mediated by nitroalkylation or *S*‐nitrosylation of proteins by modulating their functionality remains poorly understood. In the case of nitroalkylation, it has only been detected, but the type of proteins it modifies remains unknown (Aranda‐Caño et al. 2022a). Similarly, NO_2_‐FAs‐mediated *S*‐nitrosylation has been detected, but the transcription factor bZIP67 has also been identified as a target of this modification by modulating the lipid accumulation profile during embryonic development (Mata‐Pérez et al. 2020; Sánchez‐Vicente et al. 2024).

All these findings led us to further study the potential effect of NO_2_‐Ln on antioxidant proteins, in particular CAT2 protein. This antioxidant enzyme catalyzes the decomposition of H_2_O_2_ into H_2_O and O_2_ without using reducing substrates and is located in peroxisomes, an organelle where the endogenous presence of NO_2_‐Ln has been detected (Mata‐Pérez et al. 2017). The effect of NO_2_‐Ln on the enzymatic activity of recombinant CAT2 protein from Arabidopsis and in the leaf peroxisomes of the same plant species was evaluated. In both cases, NO_2_‐Ln treatment reduced CAT2 activity by 26% and 36% in leaf peroxisomes and recombinant protein, respectively (Figure 1a,b). In addition, to analyze whether this behavior was generalized to other catalases, a bovine liver recombinant CAT protein was used as a model protein in animal cells. Similarly, CAT activity was reduced by 60% after NO_2_‐Ln treatment (Figure 1c). This negative NO_2_‐Ln behavior on CAT2 activity has also been observed in other antioxidant proteins, such as recombinant APX2 protein from Arabidopsis (Aranda‐Caño et al. 2019), and even in antioxidant proteins (Tsa1) from *Saccharomyces cerevisiae* by the action of another NO_2_‐FA, such as NO_2_‐OA. These data suggest an inhibitory effect of the antioxidant function mediated by the NO_2_‐FAs present in plants and yeast.

A key component of CAT enzymatic activity is the heme group, as it allows CAT to react with H_2_O_2_ (Sharma and Ahmad 2014). In addition, the heme group of another protein, such as prostaglandin endoperoxide H synthase, has been targeted by nitro‐arachidonic acid (NO_2_‐AA). The treatment of this protein with NO_2_‐AA inhibits its activity by irreversibly disrupting heme binding to the protein (Trostchansky et al. 2011). The present study therefore investigated the hypothesis that the negative effect of NO_2_‐Ln on CAT2 activity was related to its heme group. However, treatment of the Arabidopsis recombinant CAT2 protein with NO_2_‐Ln did not change the heme group spectra compared to the control treatment (Figure S1a). This behavior was also observed for the treatment of NO_2_‐Ln with bovine liver CAT protein (Figure S1b) and with the commercial heme group (Figure S1c). Therefore, the involvement of the heme group in the enzymatic inhibition of CAT2 exerted by NO_2_‐Ln was excluded.

However, this study cannot rule out the ability of NO_2_‐FAs to release NO and to act as signaling molecules for released NO (Gorczynski et al. 2007; Mata‐Pérez et al. 2016a), such as the modulation of GSNO biosynthesis by NO_2_‐Ln (Mata‐Pérez et al. 2020). For example, released NO can exert its function mainly through protein *S*‐nitrosylation. It has been observed that CAT can be *S*‐nitrosylated by the blind docking of GSNO to Arabidopsis CAT, and Cys 420 has been identified as a putative target (Palma et al. 2020). In cadmium‐treated pea plants, peroxisomal CAT is inhibited by *S*‐nitrosylation (Ortega‐Galisteo et al. 2012). In *Ganoderma lucidum*, recombinant CAT is *S*‐nitrosylated at Cys 401, Cys 642, and Cys 653. The Cys 401 residue appears to play a key role in CAT activity (Liu et al. 2022). Thus, our second hypothesis was to investigate whether NO_2_‐Ln could regulate CAT activity through the *S*‐nitrosylation process. In the present study, it was observed that the NO_2_‐Ln treatment did not *S*‐nitrosylate the recombinant CAT2 protein. This observation serves to eliminate the possibility of NO_2_‐Ln‐dependent protein *S*‐nitrosylation being a factor in the inactivation of protein activity (Figure 2). Similar behavior was observed with the *Saccharomyces cerevisiae* Tsa1 recombinant protein treated with NO_2_‐OA, where *S*‐nitrosylation was not responsible for the inhibition of protein activity (Aranda‐Caño et al. 2022b).

NO_2_‐FAs can also modulate protein function through nitroalkylation (Aranda‐Caño et al. 2019; Aranda‐Caño et al. 2022b; Schopfer et al. 2010). Given the experimental difficulties in identifying putative nitroalkylated targets and their modified residues, this new PTM has scarcely been explored. To date, only a few works have studied NO_2_‐FAs‐mediated protein nitralkylation modification (Aranda‐Caño et al. 2019; Aranda‐Caño et al. 2022b; Fang et al. 2021; González‐Perilli et al. 2017). Given that, under the present conditions, NO_2_‐Ln did not affect the heme group or *S*‐nitrosylates CAT, it was assessed whether the inhibition of CAT activity was due to NO_2_‐Ln‐mediated nitroalkylation. The nano‐LC‐MS/MS analysis of the in vitro treatment of the recombinant CAT2 protein with NO_2_‐Ln revealed the presence of nitroalkylated peptides, with an increase of 323.43 Da corresponding to the molecular mass of NO_2_‐Ln compared to the unmodified peptides. In addition, the nitroalkylated histidine targets (46, 108, 156, 165, 201, and 248) that could potentially be responsible for the inhibition of CAT2 protein activity were identified, with His 108 and His 156 being the most highly nitroalkylated residues (Figure 3).

These results were confirmed by the docking analysis between CAT2 and NO_2_‐Ln, where only histidine residues generated feasible poses. However, the docking study ruled out His 108 as a nitroalkylation target, as it is surrounded by an environment of hydrophilic residues and a negative electrostatic potential that do not favor interaction with the NO_2_‐Ln alkyl chain (Figure S3). The fact that His 108 is located in a loop that confers a certain degree of flexibility and renders it susceptible to some degree of conformational change under in vitro analysis may provide a rationale for the high presence of this nitroalkylated residue in the mass spectrometry analyses performed after treatment of the recombinant CAT2 protein with NO_2_‐Ln. On the other hand, positive poses were detected for His 156 and His 248. However, neither His 156 nor His 248 was close to the active site or the cofactor binding site to explain the inhibitory effect on catalysis. These residues turned out to be evolutionarily conserved residues, confirming the relevance of their role in CAT. His 156 is located in the region protruding from the monomers and is involved in the interaction with upper and lower proximal monomers. His 248 is located at the interface between two monomers (Figure 6). Therefore, the binding of NO_2_‐Ln to these histidine residues affects the quaternary structure of CAT2, resulting in the inhibition of its enzymatic activity. Our results are in agreement with recent works showing that nitroalkylation reduces the activity of Arabidopsis APX2 protein, and His 43 and His 163 were detected as modified sites. These authors suggest that the modification of these residues is responsible for the inhibition of protein activity (Aranda‐Caño et al. 2019). Likewise, in *Saccharomyces cerevisiae*, the nitroalkylation of Cys 47 and Cys 171 modulates the catalytic function of Tsa1 (Aranda‐Caño et al. 2022b).

The potential role of NO_2_‐FAs in plant defense against abiotic stresses has recently been investigated. As a result, NO_2_‐Ln was found to be involved in the plant response to several environmental stresses, mainly through the induction of antioxidant and heat shock network proteins (Begara‐Morales et al. 2021; Mata‐Pérez et al. 2016c). To investigate the functional role of NO_2_‐FAs in the modulation of CAT function under abiotic stress conditions, salt stress was chosen because it is one of the conditions that most affects the growth and yield of crops and therefore increases the levels of ROS and reactive nitrogen species (RNS), leading to nitro‐oxidative stress (Begara‐Morales et al. 2014; Chaves et al. 2009; Tanou et al. 2012; Valderrama et al. 2006). CAT activity was measured in 5‐day‐old Arabidopsis cell cultures in response to salinity stress, which was induced by 150 mM NaCl for 5 min. The results obtained showed a significant increase in its activity under stress conditions (Figure 4b) to scavenge the higher levels of H_2_O_2_ induced under such circumstances (Figure 4a). Similarly, CAT activity increased in *Ganoderma lucidum* after heat stress (Liu et al. 2022), and also in the *Fragaria vulgaris* plants exposed to microcystins, which was accompanied by a rise in malondialdehyde levels (Haida et al. 2022). CAT activity can be regulated by changes in transcript abundance and consequently by changes in protein levels or by PTMs. Hence, CAT protein content was assessed and remained unchanged after salinity stress (Figure 4b), indicating that the regulation of CAT function does not appear to be due to rising protein levels. Similar behavior was observed in yeast cells, where both Tsa1 activity and H_2_O_2_ increased after heat stress, but there was no change in protein levels (Aranda‐Caño et al. 2022b).

In vitro analyses indicate that NO_2_‐Ln modulates CAT2 function through nitroalkylation. The present study therefore investigated the hypothesis that NO_2_‐FAs modulate CAT activity by nitroalkylation in vivo using salinity‐stressed arabidopsis cell cultures. Initially, the NO_2_‐Ln levels were determined in the 5‐day‐old Arabidopsis suspension cell cultures in both the control and salinity‐treated samples. The results showed that NO_2_‐Ln levels were significantly higher in response to salinity stress (Figure 5). The same results were observed in the 9‐day‐old Arabidopsis cell cultures treated with 150 mM NaCl for 5 min. However, 30 min of salinity treatment allowed higher NO_2_‐Ln levels to persist compared to the control sample, but they were lower than those detected after 5 min of salinity stress. The authors suggest a rapid response of this NO_2_‐FA to face salinity stress (Mata‐Pérez et al. 2016c).

A targeted nano‐LC‐MS/MS search of the previously identified nitroalkylated peptides in recombinant CAT2 protein was then performed to determine which target(s) were susceptible to nitroalkylation in vivo in both the control and salinity stress situations. In the control situation, only two nitroalkylated residues, His 156 and His 248 (Table 2), were identified as inhibitory to CAT2 activity because of their impact on the quaternary structure of the protein (Figure 6). In contrast, high oxidative levels generated by salinity stress may affect the nitroalkylation stability because ROS can oxidize the bond between the nucleophilic residues of the protein and NO_2_‐Ln, which would lead to the cleavage of the Michael adduct. As a result, NO_2_‐Ln is released, leading to an increase in NO_2_‐Ln levels (Figure 5), which in turn can induce the genes associated with the stress response (Mata‐Pérez et al. 2016c). Furthermore, the activity of the CAT protein, whose functionality was previously compromised by nitroalkylation, was restored to regulate the overproduction of H_2_O_2_ during salinity stress. This mechanism represents a more rapid response than that involving gene induction and the synthesis of new proteins, and could be a key element in the fine‐tuning regulation of defense mechanisms against salinity stress. The aforementioned behavior was also observed in the in vitro release of NO_2_‐FA from the Cys‐NO_2_‐FA adducts under the nitro‐oxidative conditions (Padilla et al. 2017). Furthermore, in yeast, the NO_2_‐OA‐mediated nitroalkylation of Tsa1 blocked antioxidant activity by binding NO_2_‐OA to the peroxidatic cysteine of the catalytic center. This inhibition was impaired in response to heat stress (Aranda‐Caño et al. 2022b).

**FIGURE 7:**
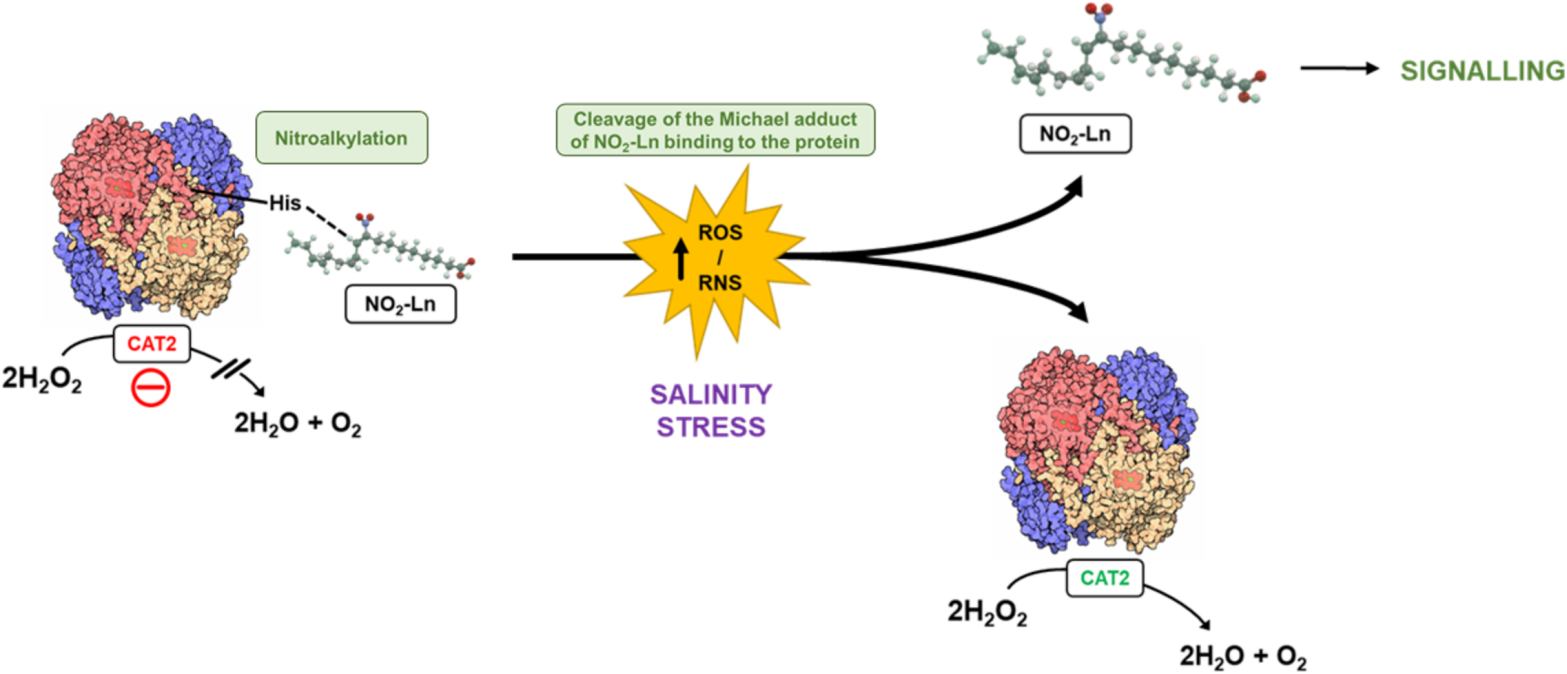
Signaling mechanism by NO_2_‐Ln‐mediated nitroalkylation of CAT2. Under physiological conditions some molecules of the catalase pool may undergo inactivation by nitroalkylation with the nitrated fatty acid with key histidine residues for the CAT2 quaternary structure which negatively interferes with its enzymatic activity. The increase in ROS and RNS levels generated by salinity stress causes oxidation and disruption of the NO_2_‐Ln binding to CAT2, resulting in the release of NO_2_‐Ln for signaling purposes and restoration of CAT2 enzymatic activity to hydrogen peroxide detoxification. CAT2 in red and green indicate non‐active and active protein, respectively.

Our data confirmed that the down‐regulation of CAT2 activity by NO_2_‐Ln was due to the in vitro and in vivo nitroalkylations of evolutionarily conserved histidines involved in the quaternary structure of CAT2. In contrast, under salinity stress, the high oxidative state could compromise the stability of the NO_2_‐Ln binding to histidine residues, leading to the oxidation of these nitroalkylated adducts, which inhibit CAT2 activity (Figure 7). This decrease in nitroalkylated CAT2 levels led to the restoration of its antioxidant functionality by suppressing the negative modulation exerted by this PTM.

All this highlights the regulatory role of nitroalkylation in protein antioxidant functionality, which is strongly influenced by the redox state and its implication in stress response processes. In summary, these findings provide a new, previously unknown perspective on the mechanisms involved in the modulation of CAT2 antioxidant activity and deepen our knowledge of nitrated fatty acids and nitric oxide signaling in plants.

## 4 MATERIALS AND METHODS

### 4.1 Plant materials, growth conditions, and salinity treatment

Suspension cultures of Arabidopsis (ecotype Columbia) cells were grown in a 200 mL of liquid growth medium based on an adaptation of the JPL solution for this plant species (Axelos et al. 1992; Jouanneau and Péaud‐Lenoël 1967). The main growth conditions were continuous shaking at 120 rpm, a constant temperature of 24°C, and continuous illumination with photosynthetically active radiation (PAR) of 50 μE^−2^ m^−2^ s^−1^ intensity. Cell cultures were subcultured every 7 days. The 5‐day‐old cell cultures were treated with 150 mM NaCl for 5 min (Fares et al. 2011; Mata‐Pérez et al. 2016c).

### 4.2 Isolation of Arabidopsis leaf peroxisomes

Arabidopsis seeds were grown in a mixture containing soil and vermiculite (2:1) in a culture chamber for 15 days (16 h light/8 h dark) and at 22/18°C. The plants were then transferred to hydroponic cultures with a suitable nutrient solution for 15 days. The nutrient solution was prepared from the macronutrient stock solution (1M KNO_3_, 1M Ca(NO_3_)_2_, 1M MgSO_4_, 1M KH_2_PO_4_, and 20 mM Fe‐EDTA) and the micronutrient stock solution (0.5M H_3_BO_3_, 0.1M MnSO_4_, 0.5M ZnSO_4_, 0.1M CuSO_4_, 0.1M Na_2_MoO_4_, and 0.1M Cl_2_Co). Stock solutions were used at the 1× concentration.

Peroxisomes were purified from the leaves of the 30‐day‐old plants grown under optimal conditions by differential centrifugation of percoll/sucrose gradients (Reumann et al. 2007). All the procedures for the isolation of Arabidopsis leaf peroxisomes were performed between 0 and 4°C.

### 4.3 Arabidopsis cell cultures crude extract preparation

The 5‐day‐old cell cultures, both control and 150 mM NaCl‐treated, were washed with water and filtered through a 150‐μm pore size membrane to remove excess NaCl. Cells were then ground to a powder in a mortar with liquid nitrogen and suspended in 1:2 (p:v) extraction buffer containing 100 mM Tris–HCl, pH 7.6, 5% sucrose, 0.1 mM EDTA, 7% polyvinylpolypyrrolidone, 0.05% Triton X‐100, 15 mM DTT, 1 mM phenylmethylsulfonyl fluoride, and 2X cocktail of protease inhibitors (Sigma‐Aldrich). The homogenates were centrifuged twice at 10,000*g* for 10 min at 4°C. The supernatant was subjected to different assays.

### 4.4 H_2_O_2_ content measurement

In the cell culture extracts, the H_2_O_2_ content was measured by a spectrophotometric assay as described by Jiang et al. (1990) with some modifications. Cell extracts were incubated in the dark at 25°C for 45 min with an assay reagent containing 500 μM ammonium ferrous sulfate, 50 mM sulfuric acid, 200 μM xylenol orange, and 200 mM sorbitol before centrifugation at 2400*g* for 10 min. The H_2_O_2_‐mediated oxidation of Fe^2+^ to Fe^3+^ was measured at 560 nm. The H_2_O_2_ concentration was calculated using a standard curve generated with commercial H_2_O_2_ (Panreac).

### 4.5 Synthesis and structural analysis of the NO_2_ ‐Ln standard and the carbon 13‐labeled nitro‐oleic acid ( ^13^C18‐NO_2_ ‐OA) internal standard

Synthesis of NO_2_‐Ln was done by nitroselenation, oxidation, and hydroselenoxide elimination, as described by Baker et al. (2005) and Aranda‐Caño et al. (2022a) with some modifications, in a tetrahydrofuran–acetonitrile mixture (1:1, v:v) dissolved in the following order: Ln (Sigma) (0.5 g, 1.79 mmol), mercury chloride (0.575 g, 1.22 mmol), phenylselenyl bromide (0.465 g, 1.97 mmol), and sodium nitrite (0.245 g, 3.55 mmol). The reaction was carried out in an argon atmosphere, and the mixture was incubated in an ice bath for 4 h. Afterwards, the other reagents were removed by filtration, and the sample was evaporated and resuspended in 12 mL of tetrahydrofuran. Later, H_2_O_2_ 33% (v/v) was added with shaking for 1 h in an ice bath, followed by extraction with hexane solvent. Next, the solvent was washed, dried, filtered, evaporated, and dissolved in a hexane/diethyl ether/acetic acid mixture (80:20:0.5, v:v:v). Then, the obtained solvent was cleaned by flash column chromatography (silica gel 60) using the previous mixture. TLC plates (Fluka Alu sheets) were used to select the fractions enriched in NO_2_‐Ln and free of Ln.

For the synthesis of the ^13^C18‐NO_2_‐OA internal standard, the above protocol was also followed. Only the incubation period of the nitroselenation process was changed, which was left for 12 h in an argon atmosphere instead of 4 h.

Finally, the synthesized compound structure was analyzed by NMR using a Bruker Avance 400 spectrometer (Billerica, MA) that operated at 400.13 MHz for 1H and 100.61 MHz for 13C.

### 4.6 Arabidopsis cell cultures lipid extraction

Lipids were extracted from the Arabidopsis cell suspension cultures, both control and treated with 150 mM NaCl, using the Bligh and Dyer method (Bligh and Dyer 1959). Afterwards, the chromatographic assay was carried out (Aranda‐Caño et al. 2022a; Aranda‐Caño et al. 2022b; Fazzari et al. 2017). To quantify loss during the acidic hydrolysis process, the obtained fractions were evaporated, dissolved in methanol, and mixed with the 10 nM internal standard ^13^C18‐NO_2_‐OA. The artificial acid‐catalyzed nitration reactions were limited by adding 250 μL of methanolic sulphanilamide (1 g/10 mL) to the samples. Next, the samples were evaporated and incubated for 1 h at 90°C with 2.5 mL of acetonitrile/hydrochloric acid (9:1). The nitrated lipids were extracted with hexane/H_2_O (2:1). Finally, the hexane fraction was selected, evaporated, dissolved in methanol, and analyzed by LC‐MS/MS.

### 4.7 Detection, identification, and quantification of endogenous NO_2_ ‐Ln from Arabidopsis cell cultures

The control and salinity‐stressed Arabidopsis cell suspension cultures were used for the detection, identification, and quantification of the amount of NO_2_‐Ln following the protocol established by Aranda‐Caño et al. (2022a). The presence of NO_2_‐Ln was confirmed by multiple reaction monitoring (MRM) by scanning with specific transitions for NO_2_‐Ln 322/46 *m*/*z* and 322/275 *m*/*z*. The quantification of NO_2_‐Ln levels was carried out using the NO_2_‐Ln standard calibration curve with 10 nM ^13^C18‐NO_2_‐OA as an internal standard.

### 4.8 Treatment with NO_2_ ‐Ln: Catalase activity assay

Arabidopsis recombinant CAT2 protein (LSBio, Life Span BioSciences), bovine liver CAT (Sigma), and leaf peroxisomes were incubated with different concentrations of NO_2_‐Ln and Ln (non‐nitrated fatty acid as a control) and methanol (as a vehicle for fatty acids) for 30 min at 25°C and 250 rpm. The determination of CAT enzyme activity was then carried out by a method based on the measurement of H_2_O_2_ disappearance at 240 nm (Aebi 1984). CAT activity was also determined in both control and 150 mM NaCl‐treated Arabidopsis suspension cell cultures.

### 4.9 Determination of CAT heme content by the pyridine hemochrome method

Arabidopsis recombinant CAT2 protein, bovine liver CAT, and the commercial hemin (Sigma) were incubated with 10 μM NO_2_‐Ln at 22°C for 60 min in the dark. For the control treatment, hemin and the recombinant proteins were incubated with methanol under the same conditions. The samples were filtered through spin desalting columns (Thermo Scientific) to remove the nitrated lipid and heme group. The protein concentration was determined by the Bradford assay. The spectrum of pyridine hemochromogen was analyzed using a spectrophotometer in the scan mode from 350 to 700 nm (Berry and Trumpower 1987; Trostchansky et al. 2011).

### 4.10 Electrophoretic methods and immunoblot analyses

Polypeptides were separated by SDS‐PAGE. Proteins were then transferred to PVDF membranes using a semi‐dry transfer system (Bio‐Rad, Hercules, CA). Protein loading was assessed by Ponceau staining of the PVDF membrane. For immunodetection, a membrane was incubated with a CAT antibody (Agrisera) diluted to 1:1000. After washing, the membrane was incubated with anti‐rabbit diluted to 1:8000. Immunoreactive bands were detected on photographic film using an enhanced ECL‐PLUS kit.

### 4.11 Biotin switch method

The recombinant CAT2 protein was incubated with 500 μM NO_2_‐Ln for 30 min at 25°C. As a positive control for the biotin switch assay, 10 μg of bovine serum albumin (BSA) was *S*‐nitrosylated with the same concentration of NO_2_‐Ln. The control treatment was performed with 500 μM Ln and methanol. The *S*‐nitrosylated samples were subjected to a biotin switch assay (Chaki et al. 2015). The non‐nitrosylated free cysteines were blocked with 30 mM methyl methane thiosulfonate (MMTS) and 2.5% SDS for 20 min at 50°C. Residual MMTS was removed by precipitation with cold acetone at −20°C. Afterwards, samples were incubated with 1 mM biotin‐HPDP and 0.1 mM ascorbate for 1 h at room temperature, and proteins were precipitated with cold acetone. Five microgram of each biotin‐labeled protein were separated by non‐reducing 10% SDS‐PAGE and transferred to a PVDF membrane (Immobilon P, Millipore, Bedford, MA). The membrane was then blocked with TBS buffer containing 1% BSA for 1.5 h. After washing, the PVDF membrane was incubated with a 1:20.000 dilution of anti‐biotin antibody for 1 h. Finally, the immunoreactive bands were detected on photographic film using an enhanced ECL‐PLUS kit (Amersham Pharmacia Biotech).

### 4.12 Nitroalkylation of Arabidopsis recombinant CAT2 protein

For the in vitro nitroalkylation treatment, Arabidopsis recombinant CAT2 protein was treated for 30 min at 25°C with slight shaking and with 350 μM NO_2_‐Ln at the 1:100 concentration ratio (CAT2:NO_2_‐Ln).

### 4.13 Treatment of the nitroalkylated recombinant CAT2 protein with H_2_O_2_

The in vitro oxidation reaction of the nitroalkylated recombinant CAT2 protein was performed according to the conditions outlined in Padilla et al. (2017). Specifically, the treatment was carried out with 1.5 mM H_2_O_2_ in phosphate buffer (50 mM, pH 7.4) at room temperature for a duration of 200 min.

### 4.14 Extraction of the in vivo*‐*nitroalkylated peptides and nitroalkylation detection by nano‐LC‐MS/MS

The extraction of the nitroalkylated peptides was carried out as described elsewhere (Aranda‐Caño et al. 2022b). Briefly, the control and salinity stress samples were precipitated with 70% cold acetone for 12 h at −20°C and centrifuged at 16,000*g* for 15 min at 4°C. The pellets of the precipitated proteins were digested with trypsin overnight at 37°C. Subsequently, samples were enriched in nitroalkylated peptides by diethyl ether extraction (1:1, v/v). The phases enriched in nitroalkylated peptides were isolated, evaporated, and resuspended in 0.1% (v/v) formic acid to give a concentration of 0.1 μg peptides/μL. After filtration through 0.2 μm filters, the samples were analyzed by nano‐LC‐MS/MS for nitroalkylation detection as described in Aranda‐Caño et al. (2022b).

### 4.15 Mass spectrometry data processing and identification of nitroalkylated proteins

The Proteome Discoverer 1.4 software (Thermo Scientific), with the SEQUEST HT search engine and the UniProt Arabidopsis thaliana database, was used to identify protein sequences from the mass spectrometry data. The search identified cysteine carbamidomethylation (+57,021 Da) as a fixed modification, and methionine oxidation (+15,995 Da), and NO_2_‐Ln‐mediated nitroalkylation (+323.43 Da) as dynamic modifications. The selected enzyme was a trypsin. It allowed up to four missing cleavage sites. The mass tolerances for the parent and fragment ions were set to 10 ppm and 0.02 Da. The Percolator tool with a 1% false discovery rate (FDR) was used to statistically confirm the results obtained. The proteins with at least three unique identified peptides were selected.

### 4.16 Relative quantification of the CAT2‐nitroalkylated peptides by nano‐LC‐MS/MS

The in vitro CAT2 nitroalkylated peptides were relatively quantified by a directed search of the peptides of interest. A strategy similar to that described in Aranda‐Caño et al. (2022b) was used. Thus, the nitroalkylated recombinant CAT2 protein with NO_2_‐Ln was used to define the *m*/*z* search windows of precursor ions and retention times (RTs) (Table S1).

The targeted search consisted of selecting the peptides containing nitroalkylation‐sensitive targets in both modified and unmodified forms to quantify the number of PSMs in each case. This analysis allowed relative quantification to identify the residues most susceptible to nitroalkylation in the recombinant Arabidopsis CAT2 protein.

The nitroalkylated residues were detected and relatively quantified in vivo using the Xcalibur program (Thermo Fisher Scientific). The *m*/*z* and RT spectrometric parameters of the peptides/precursor ions selected from the NO_2_‐Ln‐nitroalkylated recombinant CAT2 were used as nitroalkylation standards (Table S1). The MS1 bioinformatics search of both control and salinity‐stressed Arabidopsis samples was then performed, taking into account the *m*/*z* of the precursor ion with a mass tolerance of 5 ppm and 0.2 min at RT.

### 4.17 In silico studies of nitroalkylated CAT2: Coordinates preparation, docking, and molecular evolution studies

Ghemical 2.95 (Hassinen and Peräkylä 2001) was used to generate the 3D coordinates of NO_2_‐Ln and the six isomers of the nitrated form, with calculated energy values (tripos 5.2) of 11.7 kJ/mol for NO2‐Ln, 10.9 kJ/mol for both 9‐nitro‐linolenic acid (9‐NO_2_‐Ln) and 10‐nitro‐linolenic acid (10‐NO_2_‐Ln), 11.4 kJ/mol for 12‐nitro‐linolenic acid (12‐NO_2_‐Ln), 16.0 kJ/mol for 13‐nitro‐linolenic acid (13‐NO2‐Ln), 15.2 kJ/mol for 15‐nitro‐linolenic acid (15‐NO_2_‐Ln), and 10.6 kJ/mol for 16‐nitro‐linolenic acid (16‐NO_2_‐Ln).

The quaternary structure of *A. thaliana* CAT2 was modeled as previously reported (Palma et al. 2020). The molecule is very large. For the docking studies, it was truncated to obtain four sets of coordinates corresponding to one monomer (tcatA), the two interfaces between monomers (tcatAB and tcatAD) and those residues within 40 Å of the nitrogen of the imidazole ring of His 248 (tcatH248) (Figure S4). Coordinates were prepared for docking using Dock Prep, a tool implemented in Chimera (Pettersen et al. 2004), which deletes water molecules and ions, repairs truncated sidechains, adds hydrogens, and assigns partial charges.

The docking of the six isomers of NO_2_‐Ln was performed on the SwissDock server (Grosdidier et al. 2011) in the accurate mode to allow: flexibility for the side chains within 5 Å of any atom of the ligand in its reference binding mode; the possibility of not defining the region of interest (blind docking) for tcatA, tcatAB, and tcatAD; local docking, which was centered on the imidazole ring of His 240 (box size 10 × 10 × 10) for tcatH248. The results were sorted by their full‐Fitness score, a parameter that takes into account the total free energy of the system, including the solvation free energy (Zoete et al. 2010). An analysis of the results was performed using UCSF Chimera (Pettersen et al. 2004) and only those with (i) no forbidden interactions and (ii) within the 4 Å radius from the beta carbon of NO_2_‐Ln to the amino group of Lys, the thiol group of Cys, and one nitrogen of the imidazole ring of His were considered positive results. *K*_*d*_ values were estimated from the Δ*G* calculated during docking by applying the expression Δ*G* = RT ln *K*_*d*_, where *R* is the ideal gas constant (0.0019872 kcal/(mol K)) and T is the temperature (298 K).

Molecular evolution studies were conducted on the Evolutionary Trace server (Mihalek et al. 2004) using as input the model of the quaternary structure of CAT2 from *A. Thaliana*, as previously reported (Palma et al. 2020), and a BLASTP (Altschul et al. 1997) search was done on UniProtKB (The universal protein resource (UniProt) 2008) Viridiplantae, consisting of 581 sequences. Evolutionary conservation was ranked according to the rho parameter, which increases from 1 as variability (i.e., less evolutionary importance) increases (Mihalek et al. 2004).

### 4.18 Bradford

The protein concentration was analyzed by the Bradford assay using BSA as a standard curve.

### 4.19 Statistical analyses

To assess the statistical significance between means, data were analyzed using Student's *t* test. Experiments were performed at least three times with three replications per experiment. Statistically significant differences (*p* ≤ 0.05 and *p* ≤ 0.001) are indicated by asterisks.

## AUTHOR CONTRIBUTIONS

**Mounira Chaki:** Conceptualization; methodology; investigation; formal analysis; funding acquisition; writing – original draft; writing – review and editing. **Lorena Aranda‐Caño:** Conceptualization; formal analysis; writing – original draft; methodology; investigation; writing – review and editing. **Juan C. Begara‐Morales:** Methodology; investigation; formal analysis. **Beatriz Sánchez‐Calvo:** Formal analysis; methodology; investigation. **Francisco Javier López‐Jaramillo:** Data curation; supervision; software; writing – review and editing. **María N. Padilla:** Formal analysis; methodology; investigation. **Raquel Valderrama:** Formal analysis; methodology; investigation. **José Rafael Pedrajas:** Formal analysis; methodology; investigation. **Juan B. Barroso:** Funding acquisition; validation; resources; project administration; supervision; investigation; methodology; writing – review and editing; formal analysis; conceptualization.

## CONFLICT OF INTEREST STATEMENT

The authors declare no conflicts of interest.

## Supporting information


**Figure S1.** NO_2_‐Ln treatment did not modulate either the heme group of Arabidopsis recombinant CAT2 protein (a) and the bovine liver CAT protein (b) or the commercial heme (Sigma) (c). The samples were treated with control vehicle (MeOH) (black line) and with 10 μM NO_2_‐Ln (red line) for 60 min. The analysis of the spectrum of pyridine hemochromogen was carried out by the spectrophotometer in scan mode from 350 to 700 nm.
**Figure S2.** Effect of H_2_O_2_ treatment on His 108 nitroalkylation of Arabidopsis recombinant CAT2 protein by mass spectrometry. The occurrence of nitroalkylation in the recombinant CAT2 protein by NO_2_‐Ln was examined through targeted mass spectrometry techniques before and after treatment with 1.5 mM H_2_O_2_ for 200 min. The relative percentage of peptide spectral matches of the nitroalkylated and non‐nitroalkylated peptide (FSTVIHER) was determined, with His 108 serving as the target residue for nitroalkylation.
**Figure S3.** Location of His 108 of Arabidopsis catalase shown as blue spheres (a) and surface of the area surrounding His 108 (shown in green) colored by (i) the Kyte‐Doolittle scale coloring (b) from orange for the most hydrophobic to blue for the most hydrophilic and (ii) surface potential coloring (c) from blue (+10) to red (−10).
**Figure S4.** Model of the quaternary structure of catalase 2 from *A. thaliana* (a) and, in color, the truncated tcatA (b) tcatAB (c), tcatAD (d).

**Table S1.** Characterization chromatographic and spectrometric of the precursor ions that have the target residues susceptible to nitroalkylation identified in recombinant CAT2 protein treated with NO_2_‐Ln. The table presents the information of experimental peptides, mass, chemical formula, charge and time of the unmodified peptides that contain the target susceptible to nitroalkylation as the nitroalkylated peptides of that target. The nitroalkylated residues were shown in bold. His: histidine.

## Data Availability

The data that support the findings of this study are available on request from the corresponding author. The data are not publicly available due to privacy or ethical restrictions.

## References

[pro70076-bib-0001] Aebi H . Catalase in vitro. Methods in enzymology. Volume 105.Amsterdam: Elsevier; 1984. p. 121–126. 10.1016/s0076-6879(84)05016-3 6727660

[pro70076-bib-0002] Altschul SF , Madden TL , Schäffer AA , Zhang J , Zhang Z , Miller W , et al. Gapped BLAST and PSI‐BLAST: a new generation of protein database search programs. Nucleic Acids Res. 1997;25(17):3389–3402. 10.1093/nar/25.17.3389 9254694 PMC146917

[pro70076-bib-0003] Aranda‐Caño L , Sánchez‐Calvo B , Begara‐Morales JC , Chaki M , Mata‐Pérez C , Padilla MN , et al. Post‐translational modification of proteins mediated by nitro‐fatty acids in plants: Nitroalkylation. Plants Basel. 2019;8(4):82. 10.3390/plants8040082 30934982 PMC6524050

[pro70076-bib-0004] Aranda‐Caño L , Valderrama R , Chaki M , Begara‐Morales JC , Melguizo M , Barroso JB . Nitrated fatty‐acids distribution in storage biomolecules during *Arabidopsis thaliana* development. Antioxidants. 2022a;11(10):1869. 10.3390/antiox11101869 36290592 PMC9598412

[pro70076-bib-0005] Aranda‐Caño L , Valderrama R , Pedrajas JR , Begara‐Morales JC , Chaki M , Padilla MN , et al. Nitro‐oleic acid‐mediated nitroalkylation modulates the antioxidant function of cytosolic peroxiredoxin Tsa1 during heat stress in Saccharomyces cerevisiae. Antioxidants. 2022b;11(5):972. 10.3390/antiox11050972 35624836 PMC9137801

[pro70076-bib-0006] Axelos M , Curie C , Mazzolini L , Bardet C , Lescure B . A protocol for transient gene expression in Arabidopsis thaliana protoplasts isolated from cell suspension cultures. Plant Cell Rep. 1992;12(5):241–244. 10.1007/BF00237127 24197149

[pro70076-bib-0007] Baker PR , Lin Y , Schopfer FJ , Woodcock SR , Groeger AL , Batthyany C , et al. Fatty acid transduction of nitric oxide signaling: multiple nitrated unsaturated fatty acid derivatives exist in human blood and urine and serve as endogenous peroxisome proliferator‐activated receptor ligands. J Biol Chem. 2005;280(51):42464–42475. 10.1074/jbc.M504212200 16227625 PMC2266087

[pro70076-bib-0008] Baker PR , Schopfer FJ , O'Donnell VB , Freeman BA . Convergence of nitric oxide and lipid signaling: anti‐inflammatory nitro‐fatty acids. Free Radic Biol Med. 2009;46(8):989–1003. 10.1016/j.freeradbiomed.2008.11.021 19200454 PMC2761210

[pro70076-bib-0009] ` Baker PR , Schopfer FJ , Sweeney S , Freeman BA . Red cell membrane and plasma linoleic acid nitration products: synthesis, clinical identification, and quantitation. Proc Natl Acad Sci U S A. 2004;101(32):11577–11582. 10.1073/pnas.0402587101 15273286 PMC511023

[pro70076-bib-0010] Balazy M , Poff CD . Biological nitration of arachidonic acid. Curr Vasc Pharmacol. 2004;2(1):81–93. 10.2174/1570161043476465 15320836

[pro70076-bib-0011] Begara‐Morales JC , Mata‐Pérez C , Padilla MN , Chaki M , Valderrama R , Aranda‐Caño L , et al. Role of electrophilic nitrated fatty acids during development and response to abiotic stress processes in plants. J Exp Bot. 2021;72(3):917–927. 10.1093/jxb/eraa517 33161434

[pro70076-bib-0012] Begara‐Morales JC , Sánchez‐Calvo B , Chaki M , Valderrama R , Mata‐Pérez C , López‐Jaramillo J , et al. Dual regulation of cytosolic ascorbate peroxidase (APX) by tyrosine nitration and S‐nitrosylation. J Exp Bot. 2014;65(2):527–538. 10.1093/jxb/ert396 24288182 PMC3904709

[pro70076-bib-0013] Berry EA , Trumpower BL . Simultaneous determination of hemes a, b, and c from pyridine hemochrome spectra. Anal Biochem. 1987;161(1):1–15. 10.1016/0003-2697(87)90643-9 3578775

[pro70076-bib-0014] Bligh EG , Dyer WJ . A rapid method of total lipid extraction and purification. Can J Biochem Physiol. 1959;37(8):911–917. 10.1139/o59-099 13671378

[pro70076-bib-0015] Brat C , Huynh Phuoc HP , Awad O , Parmar BS , Hellmuth N , Heinicke U , et al. Endogenous anti‐tumorigenic nitro‐fatty acids inhibit the ubiquitin‐proteasome system by directly targeting the 26S proteasome. Cell Chem Biol. 2023;30(10):1277–1294. 10.1016/j.chembiol.2023.06.017 37473760

[pro70076-bib-0016] Chaki M , Shekariesfahlan A , Ageeva A , Mengel A , von Toerne C , Durner J , et al. Identification of nuclear target proteins for S‐nitrosylation in pathogen‐treated Arabidopsis thaliana cell cultures. Plant Sci. 2015;238:115–126. 10.1016/j.plantsci.2015.06.011 26259180

[pro70076-bib-0017] Chartoumpekis DV , Chen I , Salvatore SR , Schopfer FJ , Freeman BA , Khoo NKH . Adipocyte‐specific Nrf2 deletion negates nitro‐oleic acid benefits on glucose tolerance in diet‐induced obesity. Nitric Oxide. 2024;149:75–84. 10.1016/j.niox.2024.06.002 38879114

[pro70076-bib-0018] Chaves MM , Flexas J , Pinheiro C . Photosynthesis under drought and salt stress: regulation mechanisms from whole plant to cell. Ann Bot. 2009;103(4):551–560. 10.1093/aob/mcn125 18662937 PMC2707345

[pro70076-bib-0019] Chen L , Wu R , Feng J , Feng T , Wang C , Hu J , et al. Transnitrosylation mediated by the non‐canonical catalase ROG1 regulates nitric oxide signaling in plants. Dev Cell. 2020;53(4):444–457. 10.1016/j.devcel.2020.03.020 32330424

[pro70076-bib-0020] Faine LA , Cavalcanti DM , Rudnicki M , Ferderbar S , Macedo SM , Souza HP , et al. Bioactivity of nitrolinoleate: effects on adhesion molecules and CD40‐CD40L system. J Nutr Biochem. 2010;21(2):125–132. 10.1016/j.jnutbio.2008.12.004 19195864

[pro70076-bib-0021] Fang MY , Huang KH , Tu WJ , Chen YT , Pan PY , Hsiao WC , et al. Chemoproteomic profiling reveals cellular targets of nitro‐fatty acids. Redox Biol. 2021;46:102126. 10.1016/j.redox.2021.102126 34509914 PMC8441202

[pro70076-bib-0022] Fares A , Rossignol M , Peltier JB . Proteomics investigation of endogenous S‐nitrosylation in Arabidopsis. Biochem Biophys Res Commun. 2011;416(3–4):331–336. 10.1016/j.bbrc.2011.11.036 22115780

[pro70076-bib-0023] Fazzari M , Khoo NK , Woodcock SR , Jorkasky DK , Li L , Schopfer FJ , et al. Nitro‐fatty acid pharmacokinetics in the adipose tissue compartment. J Lipid Res. 2017;58(2):375–385. 10.1194/jlr.M072058 27913584 PMC5282953

[pro70076-bib-0024] Fazzari M , Trostchansky A , Schopfer FJ , Salvatore SR , Sánchez‐Calvo B , Vitturi D , et al. Olives and olive oil are sources of electrophilic fatty acid nitroalkenes. PLoS One. 2014;9(1):e84884. 10.1371/journal.pone.0084884 24454759 PMC3891761

[pro70076-bib-0025] Freeman BA , Baker PR , Schopfer FJ , Woodcock SR , Napolitano A , d'Ischia M . Nitro‐fatty acid formation and signaling. J Biol Chem. 2008;283(23):15515–15519. 10.1074/jbc.R800004200 18285326 PMC2414282

[pro70076-bib-0026] González‐Perilli L , Mastrogiovanni M , de Castro Fernandes D , Rubbo H , Laurindo F , Trostchansky A . Nitroarachidonic acid (NO(2)AA) inhibits protein disulfide isomerase (PDI) through reversible covalent adduct formation with critical cysteines. Biochim Biophys Acta Gen Subj. 2017;1861(5):1131–1139. 10.1016/j.bbagen.2017.02.013 28215702

[pro70076-bib-0027] Gorczynski MJ , Huang J , King SB . Regio‐ and stereospecific syntheses and nitric oxide donor properties of (E)‐9‐ and (E)‐10‐nitrooctadec‐9‐enoic acids. Org Lett. 2006;8(11):2305–2308. 10.1021/ol060548w 16706512 PMC3481162

[pro70076-bib-0028] Gorczynski MJ , Huang J , Lee H , King SB . Evaluation of nitroalkenes as nitric oxide donors. Bioorg Med Chem Lett. 2007;17(7):2013–2017. 10.1016/j.bmcl.2007.01.016 17270440

[pro70076-bib-0029] Grosdidier A , Zoete V , Michielin O . SwissDock, a protein‐small molecule docking web service based on EADock DSS. Nucleic Acids Res. 2011;39:W270–W277. 10.1093/nar/gkr366 21624888 PMC3125772

[pro70076-bib-0030] Haida M , El Khalloufi F , Mugani R , Redouane EM , Campos A , Vasconcelos V , et al. Effects of irrigation with microcystin‐containing water on growth, physiology, and antioxidant defense in strawberry *Fragaria vulgaris* under hydroponic culture. Toxins Basel. 2022;14(3):198. 10.3390/toxins14030198 35324694 PMC8950678

[pro70076-bib-0031] Hassinen T , Peräkylä M . New energy terms for reduced protein models implemented in an off‐lattice force field. J Comput Chem. 2001;22(12):1229–1242.

[pro70076-bib-0032] Jiang ZY , Woollard AC , Wolff SP . Hydrogen peroxide production during experimental protein glycation. FEBS Lett. 1990;268(1):69–71. 10.1016/0014-5793(90)80974-n 2384174

[pro70076-bib-0033] Jouanneau J‐P , Péaud‐Lenoël C . Growth and synthesis of proteins in cell suspensions of a kinetin dependent tobacco. Physiol Plant. 1967;20:834–850.

[pro70076-bib-0034] Khoo NKH , Schopfer FJ . Nitrated fatty acids: from diet to disease. Curr Opin Physio. 2019;9:67–72. 10.1016/j.cophys.2019.04.013 PMC6785038 31598569

[pro70076-bib-0035] Li Y , Zhang J , Schopfer FJ , Martynowski D , Garcia‐Barrio MT , Kovach A , et al. Molecular recognition of nitrated fatty acids by PPAR gamma. Nat Struct Mol Biol. 2008;15(8):865–867. 10.1038/nsmb.1447 18604218 PMC2538624

[pro70076-bib-0036] Lin YW . Structure and function of heme proteins regulated by diverse post‐translational modifications. Arch Biochem Biophys. 2018;641:1–30. 10.1016/j.abb.2018.01.009 29407792

[pro70076-bib-0037] Liu R , Zhu T , Chen X , Wang Z , Yang Z , Ren A , et al. GSNOR regulates ganoderic acid content in *Ganoderma lucidum* under heat stress through S‐nitrosylation of catalase. Commun Biol. 2022;5(1):32. 10.1038/s42003-021-02988-0 35017648 PMC8752759

[pro70076-bib-0038] Mata‐Pérez C , Padilla MN , Sánchez‐Calvo B , Begara‐Morales JC , Valderrama R , Chaki M , et al. Endogenous biosynthesis of S‐nitrosoglutathione from nitro‐fatty acids in plants. Front Plant Sci. 2020;11:962. 10.3389/fpls.2020.00962 32714353 PMC7340149

[pro70076-bib-0039] Mata‐Pérez C , Sánchez‐Calvo B , Begara‐Morales JC , Carreras A , Padilla MN , Melguizo M , et al. Nitro‐linolenic acid is a nitric oxide donor. Nitric Oxide. 2016a;57:57–63. 10.1016/j.niox.2016.05.003 27164295

[pro70076-bib-0040] Mata‐Pérez C , Sánchez‐Calvo B , Begara‐Morales JC , Padilla MN , Valderrama R , Corpas FJ , et al. Nitric oxide release from nitro‐fatty acids in Arabidopsis roots. Plant Signal Behav. 2016b;11(3):e1154255. 10.1080/15592324.2016.1154255 26910757 PMC4883945

[pro70076-bib-0041] Mata‐Pérez C , Sánchez‐Calvo B , Padilla MN , Begara‐Morales JC , Luque F , Melguizo M , et al. Nitro‐fatty acids in plant signaling: nitro‐linolenic acid induces the molecular chaperone network in Arabidopsis. Plant Physiol. 2016c;170(2):686–701. 10.1104/pp.15.01671 26628746 PMC4734579

[pro70076-bib-0042] Mata‐Pérez C , Sánchez‐Calvo B , Padilla MN , Begara‐Morales JC , Valderrama R , Corpas FJ , et al. Nitro‐fatty acids in plant signaling: new key mediators of nitric oxide metabolism. Redox Biol. 2017;11:554–561. 10.1016/j.redox.2017.01.002 28104576 PMC5241575

[pro70076-bib-0043] Mhamdi A , Queval G , Chaouch S , Vanderauwera S , Van Breusegem F , Noctor G . Catalase function in plants: a focus on Arabidopsis mutants as stress‐mimic models. J Exp Bot. 2010;61(15):4197–4220. 10.1093/jxb/erq282 20876333

[pro70076-bib-0044] Mihalek I , Res I , Lichtarge O . A family of evolution‐entropy hybrid methods for ranking protein residues by importance. J Mol Biol. 2004;336(5):1265–1282. 10.1016/j.jmb.2003.12.078 15037084

[pro70076-bib-0045] Munns R , Tester M . Mechanisms of salinity tolerance. Annu Rev Plant Biol. 2008;59:651–681. 10.1146/annurev.arplant.59.032607.092911 18444910

[pro70076-bib-0046] Ortega‐Galisteo AP , Rodríguez‐Serrano M , Pazmiño DM , Gupta DK , Sandalio LM , Romero‐Puertas MC . S‐nitrosylated proteins in pea (*Pisum sativum* L.) leaf peroxisomes: changes under abiotic stress. J Exp Bot. 2012;63(5):2089–2103. 10.1093/jxb/err414 22213812 PMC3295397

[pro70076-bib-0047] Padilla MN , Mata‐Pérez C , Melguizo M , Barroso JB . In vitro nitro‐fatty acid release from Cys‐NO(2)‐fatty acid adducts under nitro‐oxidative conditions. Nitric Oxide. 2017;68:14–22. 10.1016/j.niox.2016.12.009 28030780

[pro70076-bib-0048] Padovani D , Hessani A , Castillo FT , Liot G , Andriamihaja M , Lan A , et al. Sulfheme formation during homocysteine S‐oxygenation by catalase in cancers and neurodegenerative diseases. Nat Commun. 2016;7:13386. 10.1038/ncomms13386 27848965 PMC5116089

[pro70076-bib-0049] Palma JM , Mateos RM , López‐Jaramillo J , Rodríguez‐Ruiz M , González‐Gordo S , Lechuga‐Sancho AM , et al. Plant catalases as NO and H(2)S targets. Redox Biol. 2020;34:101525. 10.1016/j.redox.2020.101525 32505768 PMC7276441

[pro70076-bib-0050] Pettersen EF , Goddard TD , Huang CC , Couch GS , Greenblatt DM , Meng EC , et al. UCSF Chimera—a visualization system for exploratory research and analysis. J Comput Chem. 2004;25(13):1605–1612. 10.1002/jcc.20084 15264254

[pro70076-bib-0051] Reumann S , Babujee L , Ma C , Wienkoop S , Siemsen T , Antonicelli GE , et al. Proteome analysis of Arabidopsis leaf peroxisomes reveals novel targeting peptides, metabolic pathways, and defense mechanisms. Plant Cell. 2007;19(10):3170–3193. 10.1105/tpc.107.050989 17951448 PMC2174697

[pro70076-bib-0052] Rom O , Liu Y , Chang L , Chen YE , Aviram M . Editorial: Nitro‐fatty acids: novel drug candidates for the co‐treatment of atherosclerosis and nonalcoholic fatty liver disease. Curr Opin Lipidol. 2020;31(2):104–107. 10.1097/mol.0000000000000666 32132415 PMC7534545

[pro70076-bib-0053] Rudolph V , Schopfer FJ , Khoo NK , Rudolph TK , Cole MP , Woodcock SR , et al. Nitro‐fatty acid metabolome: saturation, desaturation, beta‐oxidation, and protein adduction. J Biol Chem. 2009;284(3):1461–1473. 10.1074/jbc.M802298200 19015269 PMC2615530

[pro70076-bib-0054] Sánchez‐Calvo B , Cassina A , Rios N , Peluffo G , Boggia J , Radi R , et al. Nitro‐arachidonic acid prevents angiotensin II‐induced mitochondrial dysfunction in a cell line of kidney proximal tubular cells. PLoS One. 2016;11(3):e0150459. 10.1371/journal.pone.0150459 26943326 PMC4778875

[pro70076-bib-0055] Sánchez‐Vicente I , Albertos P , Sanz C , Wybouw B , De Rybel B , Begara‐Morales JC , et al. Reversible S‐nitrosylation of bZIP67 by peroxiredoxin IIE activity and nitro‐fatty acids regulates the plant lipid profile. Cell Rep. 2024;43(4):114091. 10.1016/j.celrep.2024.114091 38607914 PMC11063630

[pro70076-bib-0056] Schopfer FJ , Cipollina C , Freeman BA . Formation and signaling actions of electrophilic lipids. Chem Rev. 2011;111(10):5997–6021. 10.1021/cr200131e 21928855 PMC3294277

[pro70076-bib-0057] Schopfer FJ , Cole MP , Groeger AL , Chen CS , Khoo NK , Woodcock SR , et al. Covalent peroxisome proliferator‐activated receptor gamma adduction by nitro‐fatty acids: selective ligand activity and anti‐diabetic signaling actions. J Biol Chem. 2010;285(16):12321–12333. 10.1074/jbc.M109.091512 20097754 PMC2852971

[pro70076-bib-0058] Schopfer FJ , Khoo NKH . Nitro‐fatty acid logistics: formation, biodistribution, signaling, and pharmacology. Trends Endocrinol Metab. 2019;30(8):505–519. 10.1016/j.tem.2019.04.009 31196614 PMC7121905

[pro70076-bib-0059] Sharma I , Ahmad P . Catalase: a versatile antioxidant in plants. Oxidative damage to plants. Amsterdam: Elsevier; 2014. p. 131–148. 10.1016/B978-0-12-799963-0.00004-6

[pro70076-bib-0060] Siddiqui MH , Al‐Whaibi MH , Basalah MO . Role of nitric oxide in tolerance of plants to abiotic stress. Protoplasma. 2011;248(3):447–455. 10.1007/s00709-010-0206-9 20827494

[pro70076-bib-0061] Tanou G , Filippou P , Belghazi M , Job D , Diamantidis G , Fotopoulos V , et al. Oxidative and nitrosative‐based signaling and associated post‐translational modifications orchestrate the acclimation of citrus plants to salinity stress. Plant J. 2012;72(4):585–599. 10.1111/j.1365-313X.2012.05100.x 22780834

[pro70076-bib-0062] The UniProt Consortium . The Universal Protein Resource (UniProt). Nucleic Acids Res. 2008;36:D190–D195. 10.1093/nar/gkm895 18045787 PMC2238893

[pro70076-bib-0063] Trostchansky A , Bonilla L , Thomas CP , O'Donnell VB , Marnett LJ , Radi R , et al. Nitroarachidonic acid, a novel peroxidase inhibitor of prostaglandin endoperoxide H synthases 1 and 2. J Biol Chem. 2011;286(15):12891–12900. 10.1074/jbc.M110.154518 21266582 PMC3075636

[pro70076-bib-0064] Tsikas D , Zoerner AA , Jordan J . Oxidized and nitrated oleic acid in biological systems: analysis by GC‐MS/MS and LC‐MS/MS, and biological significance. Biochim Biophys Acta. 2011;1811(11):694–705. 10.1016/j.bbalip.2011.06.015 21771665

[pro70076-bib-0065] Valderrama R , Corpas FJ , Carreras A , Fernández‐Ocaña A , Chaki M , Luque F , et al. Nitrosative stress in plants. FEBS Lett. 2007;581(3):453–461. 10.1016/j.febslet.2007.01.006 17240373

[pro70076-bib-0066] Valderrama R , Corpas FJ , Carreras A , Gómez‐Rodríguez MV , Chaki M , Pedrajas JR , et al. The dehydrogenase‐mediated recycling of NADPH is a key antioxidant system against salt‐induced oxidative stress in olive plants. Plant Cell Environ. 2006;29(7):1449–1459. 10.1111/j.1365-3040.2006.01530.x 17080966

[pro70076-bib-0067] Vollár M , Feigl G , Oláh D , Horváth A , Molnár Á , Kúsz N , et al. Nitro‐oleic acid in seeds and differently developed seedlings of *Brassica napus* L. Plants Basel. 2020;9(3):406. 10.3390/plants9030406 32214020 PMC7154869

[pro70076-bib-0068] Wang P , Killeen ME , Sumpter TL , Ferris LK , Falo LD Jr , Freeman BA , et al. Electrophilic nitro‐fatty acids suppress psoriasiform dermatitis: STAT3 inhibition as a contributory mechanism. Redox Biol. 2021;43:101987. 10.1016/j.redox.2021.101987 33946017 PMC8111320

[pro70076-bib-0069] Wang W , Li C , Yang T . Protection of nitro‐fatty acid against kidney diseases. Am J Physiol Renal Physiol. 2016;310(8):F697–F704. 10.1152/ajprenal.00321.2015 26719362 PMC4835926

[pro70076-bib-0070] Willekens H , Chamnongpol S , Davey M , Schraudner M , Langebartels C , Van Montagu M , et al. Catalase is a sink for H_2_O_2_ and is indispensable for stress defence in C3 plants. EMBO J. 1997;16(16):4806–4816. 10.1093/emboj/16.16.4806 9305623 PMC1170116

[pro70076-bib-0071] Zhang M , Barg R , Yin M , Gueta‐Dahan Y , Leikin‐Frenkel A , Salts Y , et al. Modulated fatty acid desaturation via overexpression of two distinct omega‐3 desaturases differentially alters tolerance to various abiotic stresses in transgenic tobacco cells and plants. Plant J. 2005;44(3):361–371. 10.1111/j.1365-313X.2005.02536.x 16236147

[pro70076-bib-0072] Zhao CB , Chen WB , Wang WZ , Gong FX , Fan CQ , Li Y , et al. Nitro‐oleic acid ameliorates erectile dysfunction in a streptozotocin‐induced rat model of diabetes by inhibiting oxidative stress and apoptosis and activating the NO/cGMP pathway. Asian J Androl. 2024;26(1):57–66. 10.4103/aja202331 37695220 PMC10846833

[pro70076-bib-0073] Zhou C , Su M , Sun P , Tang X , Yin KJ . Nitro‐oleic acid‐mediated blood‐brain barrier protection reduces ischemic brain injury. Exp Neurol. 2021;346:113861. 10.1016/j.expneurol.2021.113861 34499902

[pro70076-bib-0074] Zoete V , Grosdidier A , Cuendet M , Michielin O . Use of the FACTS solvation model for protein‐ligand docking calculations. Application to EADock. J Mol Recognit. 2010;23(5):457–461. 10.1002/jmr.1012 20101644

