# Supplementary material for: Nitro‐fatty acids‐mediated nitroalkylation modulates fine‐tuning catalase antioxidant function during salinity stress in plants
Source: Protein Sci. 2025 Feb 25;34(3):e70076. doi: 10.1002/pro.70076 (PMC11862108; doi:10.1002/pro.70076)
Supplement: Supplementary file 1 — Figure S1. NO2‐Ln treatment did not modulate either the heme group of Arabidopsis recombinant CAT2 protein (a) and the bovine liver CAT protein (b) or the commercial heme (Sigma) (c). The samples were treated with control vehicle (MeOH) (black line) and with 10 μM NO2‐Ln (red line) for 60 min. The analysis of the spectrum of pyridine hemochromogen was carried out by the spectrophotometer in scan mode from 350 to 700 nm. Figure S2. Effect of H2O2 treatment on His 108 nitroalkylation of Arabidopsis recombinant CAT2 protein by mass spectrometry. The occurrence of nitroalkylation in the recombinant CAT2 protein by NO2‐Ln was examined through targeted mass spectrometry techniques before and after treatment with 1.5 mM H2O2 for 200 min. The relative percentage of peptide spectral matches of the nitroalkylated and non‐nitroalkylated peptide (FSTVIHER) was determined, with His 108 serving as the target residue for nitroalkylation. Figure S3. Location of His 108 of Arabidopsis catalase shown as blue spheres (a) and surface of the area surrounding His 108 (shown in green) colored by (i) the Kyte‐Doolittle scale coloring (b) from orange for the most hydrophobic to blue for the most hydrophilic and (ii) surface potential coloring (c) from blue (+10) to red (−10). Figure S4. Model of the quaternary structure of catalase 2 from A. thaliana (a) and, in color, the truncated tcatA (b) tcatAB (c), tcatAD (d). [file PRO-34-e70076-s002.pptx]

## Slide 1
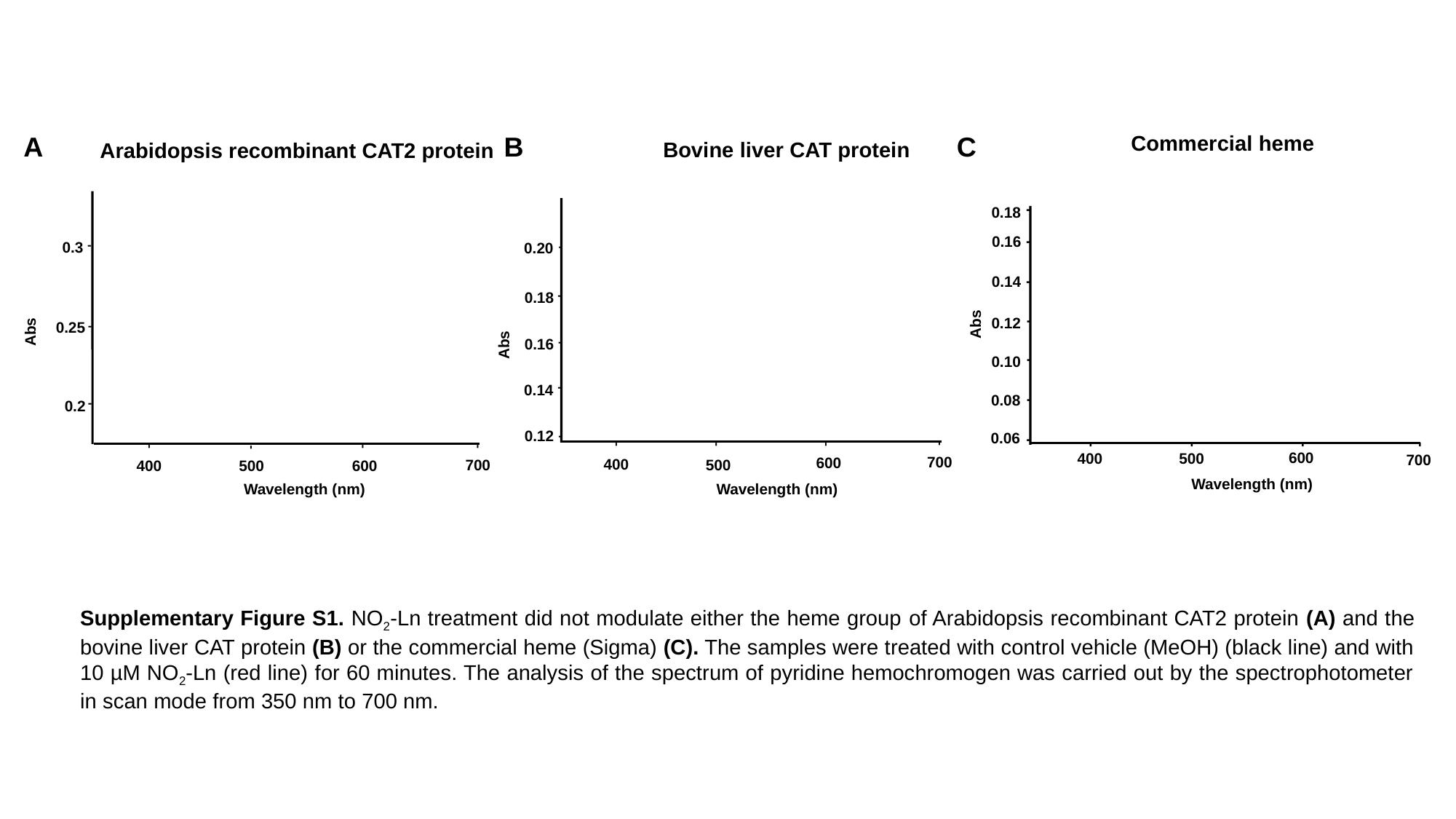

A
B
C
Commercial heme
Bovine liver CAT protein
Arabidopsis recombinant CAT2 protein
-
0.3
-
Abs
0.25
-
0.2
-
-
-
-
700
600
400
500
Wavelength (nm)
-
0.18
-
0.16
-
0.14
Abs
-
0.12
-
0.10
-
0.08
0.06
-
-
-
-
-
600
400
500
700
Wavelength (nm)
-
0.20
-
0.18
Abs
-
0.16
-
0.14
0.12
-
-
-
-
-
700
600
400
500
Wavelength (nm)
Supplementary Figure S1. NO2-Ln treatment did not modulate either the heme group of Arabidopsis recombinant CAT2 protein (A) and the bovine liver CAT protein (B) or the commercial heme (Sigma) (C). The samples were treated with control vehicle (MeOH) (black line) and with 10 µM NO2-Ln (red line) for 60 minutes. The analysis of the spectrum of pyridine hemochromogen was carried out by the spectrophotometer in scan mode from 350 nm to 700 nm.

## Slide 2
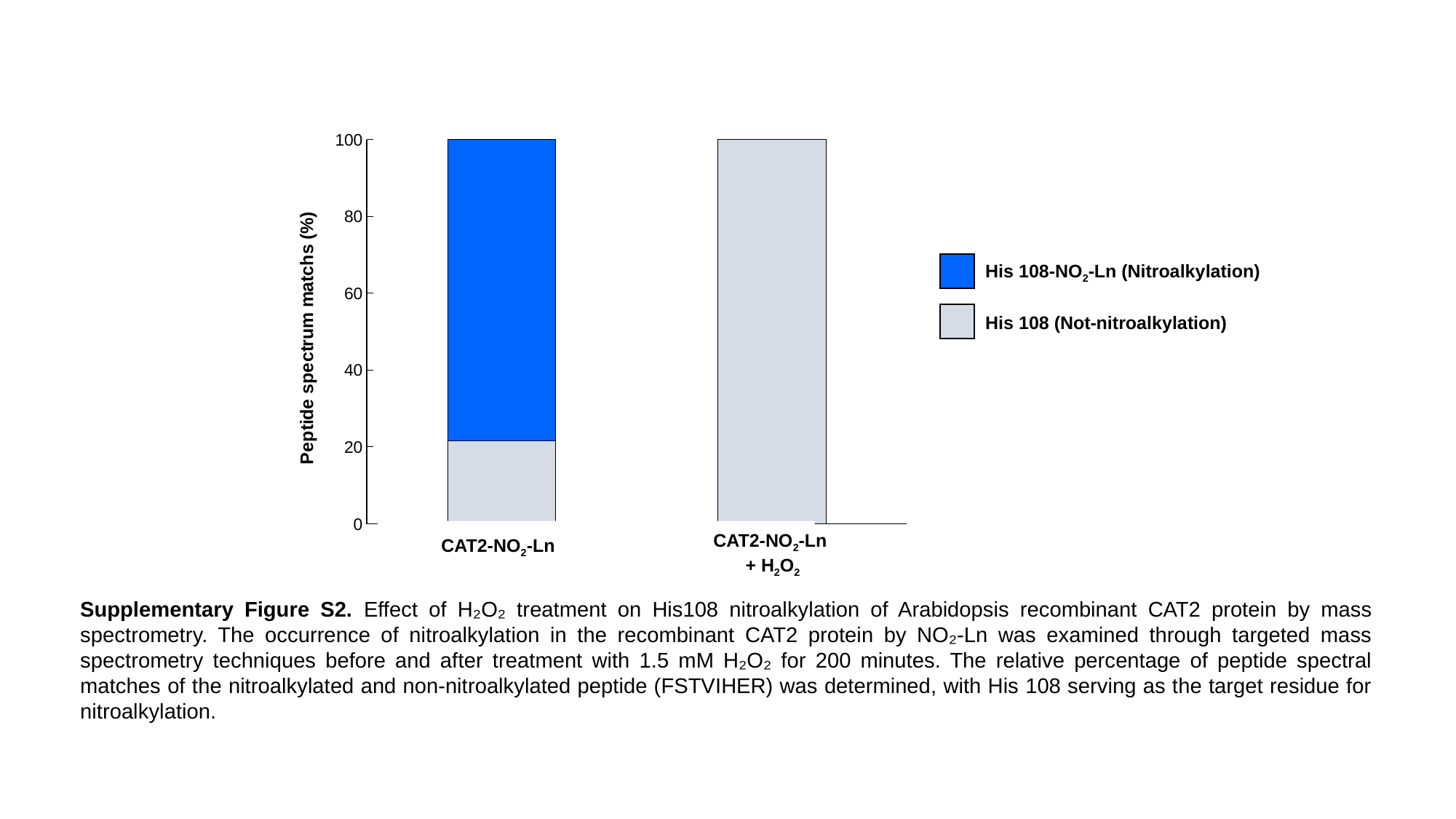

### Chart
| Category | Non-nitroalkylated His 108 | Nitroalkylated His 108 |
|---|---|---|
| CONTROL | 21.674214146156 | 78.325785853844 |
| h2o2 | 100.0 | 0.0 |
CAT2-NO2-Ln
 + H2O2
CAT2-NO2-Ln
His 108-NO2-Ln (Nitroalkylation)
His 108 (Not-nitroalkylation)
Supplementary Figure S2. Effect of H₂O₂ treatment on His108 nitroalkylation of Arabidopsis recombinant CAT2 protein by mass spectrometry. The occurrence of nitroalkylation in the recombinant CAT2 protein by NO₂-Ln was examined through targeted mass spectrometry techniques before and after treatment with 1.5 mM H₂O₂ for 200 minutes. The relative percentage of peptide spectral matches of the nitroalkylated and non-nitroalkylated peptide (FSTVIHER) was determined, with His 108 serving as the target residue for nitroalkylation.

## Slide 3
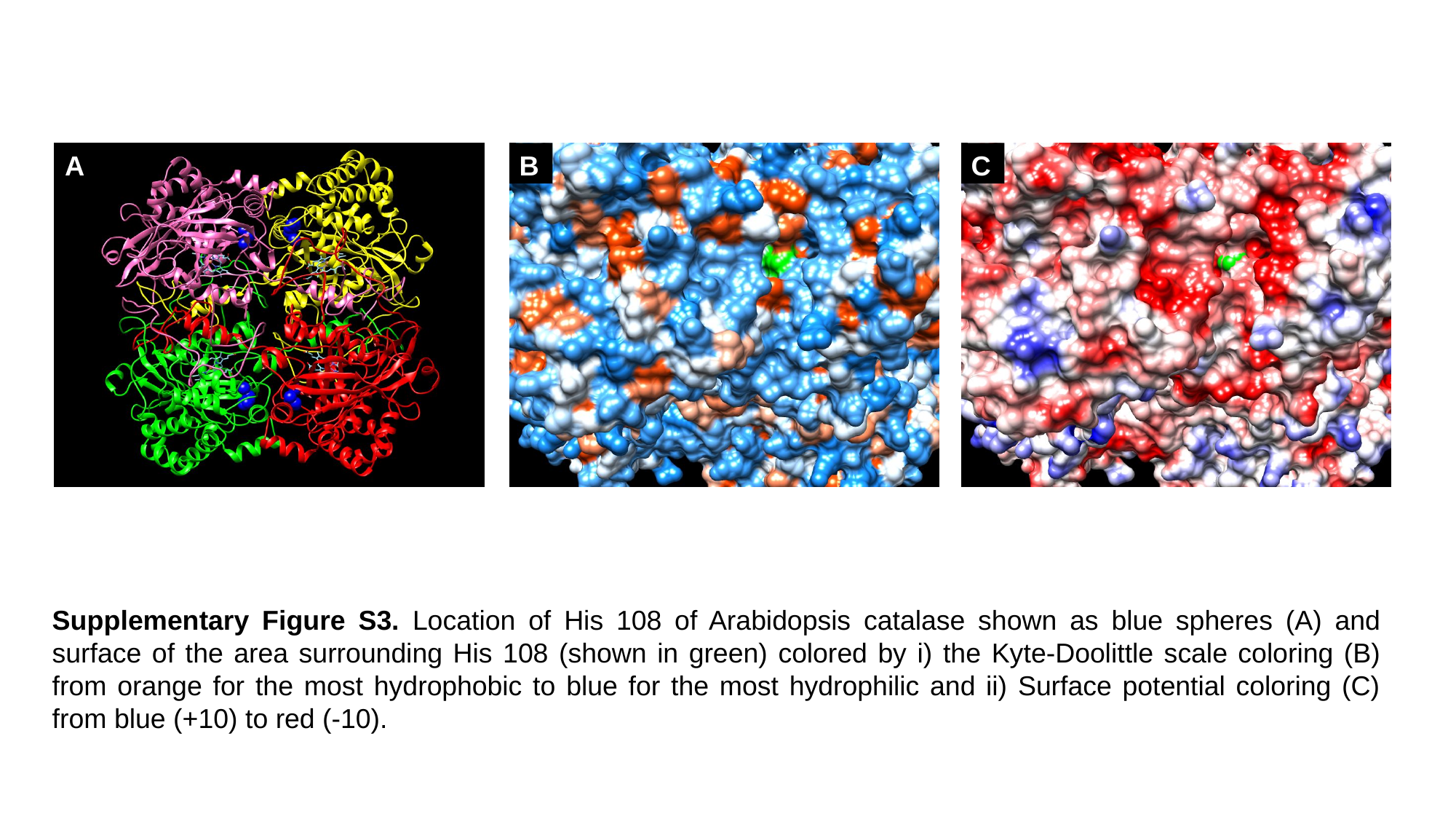

A
B
C
Supplementary Figure S3. Location of His 108 of Arabidopsis catalase shown as blue spheres (A) and surface of the area surrounding His 108 (shown in green) colored by i) the Kyte-Doolittle scale coloring (B) from orange for the most hydrophobic to blue for the most hydrophilic and ii) Surface potential coloring (C) from blue (+10) to red (-10).

## Slide 4
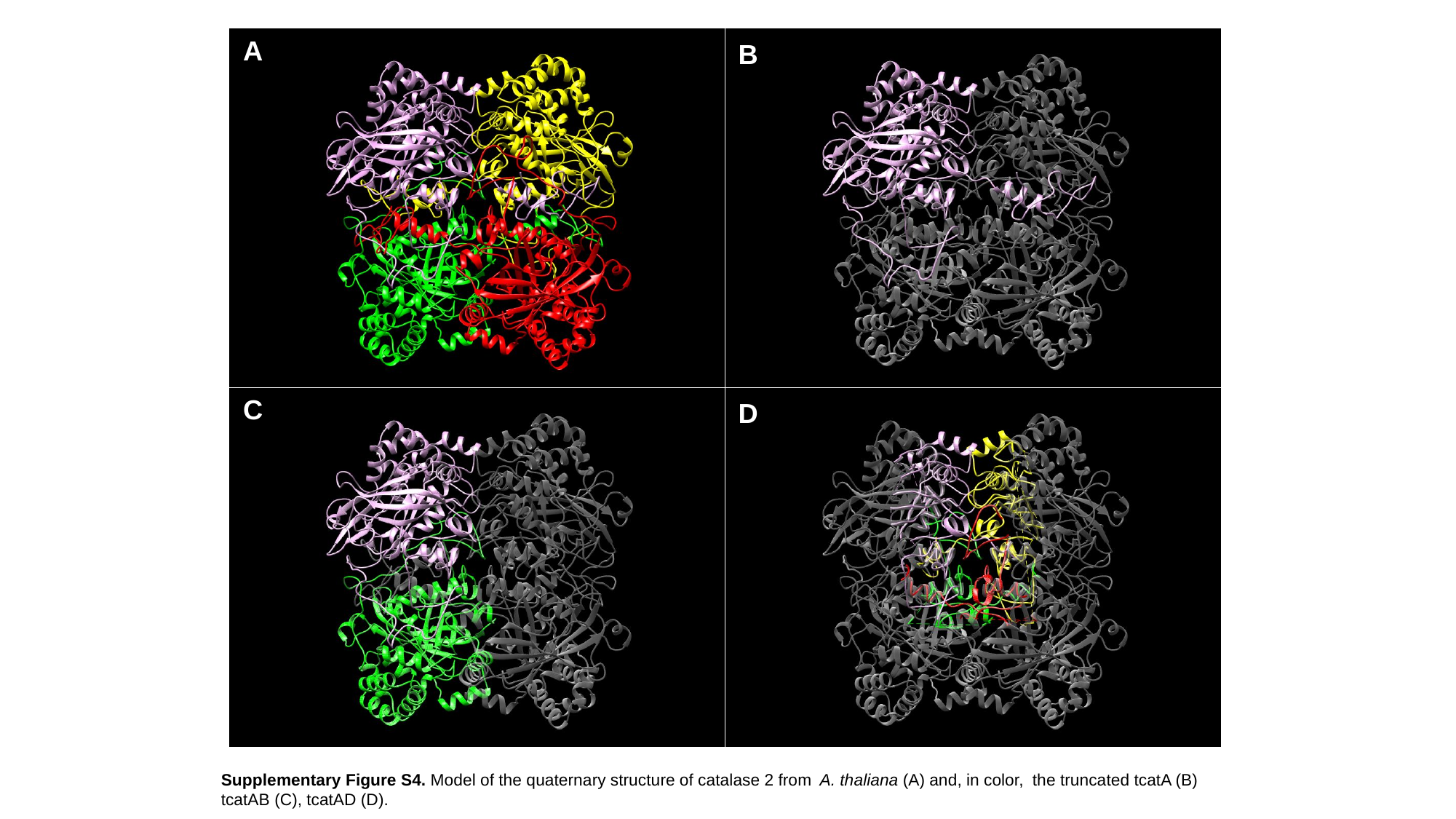

A
C
B
D
Supplementary Figure S4. Model of the quaternary structure of catalase 2 from A. thaliana (A) and, in color, the truncated tcatA (B) tcatAB (C), tcatAD (D).
