# Supplementary material for: Nitro‐fatty acids‐mediated nitroalkylation modulates fine‐tuning catalase antioxidant function during salinity stress in plants
Source: Protein Sci. 2025 Feb 25;34(3):e70076. doi: 10.1002/pro.70076 (PMC11862108; doi:10.1002/pro.70076)
Supplement: Supplementary file 2 — Table S1. Characterization chromatographic and spectrometric of the precursor ions that have the target residues susceptible to nitroalkylation identified in recombinant CAT2 protein treated with NO2‐Ln. The table presents the information of experimental peptides, mass, chemical formula, charge and time of the unmodified peptides that contain the target susceptible to nitroalkylation as the nitroalkylated peptides of that target. The nitroalkylated residues were shown in bold. His: histidine. [file PRO-34-e70076-s001.docx]

**Supplementary Table SI.** Characterization chromatographic and spectrometric of the precursor ions that have the target residues susceptible to nitroalkylation identified in recombinant CAT2 protein treated with NO_2_-Ln. The table present the information of experimental peptides, mass, chemical formula, charge and time of the unmodified peptides that contain the target susceptible to nitroalkylation as the nitroalkylated peptides of that target. The nitroalkylated residues were shown in bold. His: histidine.

| **Target residues** | **Experimental peptides** | **Mass/charge [m/z]** | **Chemical formula [M]** | **Charge** | **Start time [min]** | **End time [min]** |
| --- | --- | --- | --- | --- | --- | --- |
| **His 46** | GPILLEDYHLVEK | 50.928.021 | C71H112N16O21 | +3 | 29.00 | 44.00 |
|  | GPILLEDY**H**LVEK | 61.701.676 | C89H141N17O25 | +3 | 54.00 | 72.00 |
| **His 108** | FSTVIHER | 49.476.417 | C44H69N13O13 | +2 | 10.00 | 25.00 |
|  | FSTVI**H**ER | 43.791.509 | C62H98N14O17 | +3 | 44.00 | 62.00 |
| **His 156** | DGMKFPDMVHALKPNPK | 96.299.223 | C86H137N23O23S2 | +2 | 27.00 | 37.00 |
|  | DGmKFPDmVHALKPNPK | 49.799.467 | C86H137N23O27S2 | +4 | 15.00 | 25.00 |
|  | DGmKFPDmVHALKPNPK | 48.999.721 | C86H137N23O25S2 | +4 | 15.00 | 25.00 |
|  | DGMkFPDMV**H**ALKPNPK | 75.006.713 | C104H166N24O27S2 | +3 | 17.00 | 27.00 |
|  | DGMKFPDmVHALKPNPK | 64.766.222 | C86H137N23O24S2 | +3 | 19.00 | 29.00 |
|  | FPDMVHALKPNPK | 49.860.263 | C69H108N18O17S | +3 | 18.00 | 33.00 |
|  | FPDMV**H**ALKPNPK | 45.500.620 | C87H137N19O21S | +4 | 45.00 | 61.00 |
|  | FPDMV**H**ALKPNPK | 92.500.005 | C87H137N19O23S | +2 | 45.00 | 55.00 |
|  | FPDmV**H**ALKPNPK | 91.700.259 | C87H137N19O22S | +2 | 45.00 | 55.00 |
|  | FPDmVHALKPNPK | 50.926.590 | C69H108N18O19S | +3 | 14.00 | 24.00 |
|  | FPDmVHALKPNPK | 50.393.427 | C69H108N18O18S | +3 | 14.00 | 24.00 |
| **His 165** | SHIQENWR | 53.526.232 | C46H68N16O14 | +2 | 8.00 | 23.00 |
|  | S**H**IQENWR | 69.686.715 | C64H97N17O18 | +2 | 44.00 | 61.00 |
| **His 201** | HMDGSGVNTYMLINK | 56.060.085 | C71H114N20O23S2 | +3 | 22.00 | 37.00 |
|  | HmDGSGVNTYmLINK | 87.238.746 | C71H114N20O27S2 | +2 | 21.00 | 34.00 |
|  | HmDGSGVNTYmLINK | 85.639.255 | C71H114N20O25S2 | +2 | 21.00 | 34.00 |
|  | **H**MDGSGVNTYMLINK | 66.833.740 | C89H143N21O27S2 | +3 | 54.00 | 71.00 |
|  | **H**MDGSGVNTYmLINK | 103.399.229 | C89H143N21O31S2 | +2 | 54.00 | 65.00 |
|  | **H**mDGSGVNTYmLINK | 101.799.738 | C89H143N21O29S2 | +2 | 54.00 | 65.00 |
|  | **H**mDGSGVNTYMLINK | 100.999.992 | C89H143N21O28S2 | +2 | 54.00 | 65.00 |
|  | HmDGSGVNTYMLINK | 84.839.509 | C71H114N20O24S2 | +2 | 24.00 | 34.00 |
| **His 248** | VGGTNHSHATQDLYDSIAAGNYPEWK | 70.858.095 | C124H179N35O42 | +4 | 27.00 | 42.00 |
|  | VGGTNHS**H**ATQDLYDSIAAGNYPEWK | 78.938.336 | C142H208N36O46 | +4 | 47.00 | 62.00 |
